# Large, heterometallic coordination cages based on ditopic metallo-ligands with 3-pyridyl donor groups†
†Electronic supplementary information (ESI) available. CCDC 1024735–1024740. For ESI and crystallographic data in CIF or other electronic format see DOI: 10.1039/c4sc03046j



**DOI:** 10.1039/c4sc03046j

**Published:** 2014-11-11

**Authors:** Matthew D. Wise, Julian J. Holstein, Philip Pattison, Celine Besnard, Euro Solari, Rosario Scopelliti, Gerard Bricogne, Kay Severin

**Affiliations:** a Institut des Sciences et Ingénierie Chimiques , Ecole Polytechnique Fédérale de Lausanne (EPFL) , 1015 Lausanne , Switzerland . Email: kay.severin@epfl.ch ; Fax: +41 21-693-9305; b GZG , Abteilung Kristallographie , Georg-August-Universität Göttingen , Goldschmidtstr. 1 , 37077 Göttingen , Germany; c Swiss-Norwegian Beamline , ESRF , F-38043 Grenoble , Switzerland; d Laboratory of Crystallography , Ecole Polytechnique Fédérale de Lausanne (EPFL) , 1015 Lausanne , Switzerland; e Laboratory of X-ray Crystallography , University of Geneva , CH-1211-Geneva 4 , Switzerland; f Global Phasing Ltd. , Sheraton House , Castle Park Cambridge CB3 0AX , England

## Abstract

Ditopic N-donor ligands with terminal 4-pyridyl groups are omnipresent in coordination-based self-assembly. The utilization of ligands with 3-pyridyl donor groups is significantly less common, because the intrinsic conformational flexibility of these ligands tends to favor the formation of small aggregates. Here, we show that large Pd_6_L_12_^12+^ cages can be obtained by reaction of Pd(ii) salts with metallo-ligands L bearing terminal 3-pyridyl groups. The easy-to-access metallo-ligands contain an Fe(ii) clathrochelate core. These sterically demanding clathrochelate complexes prevent the formation of smaller aggregates, which is observed for less bulky analogous building blocks. The cages were shown to bind BF_4_^–^ and BPh_4_^–^ anions in aqueous solvent mixtures, whilst the lateral size of the clathrochelate significantly affects their guest encapsulation behavior.

## Abstract 2

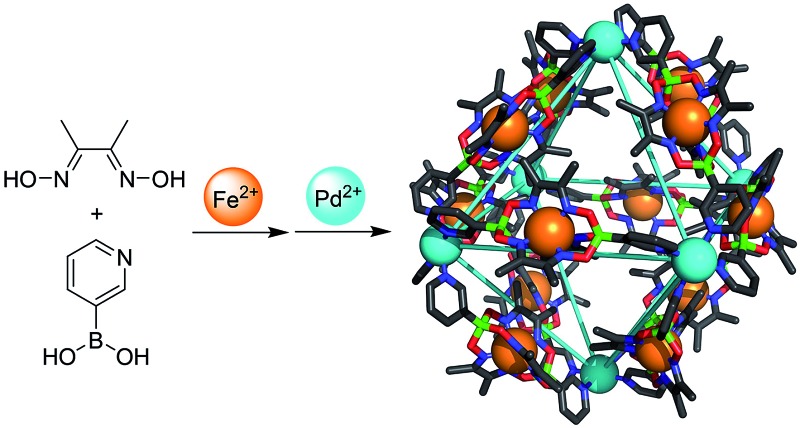
The two-step synthesis of large, heterometallic coordination cages based on ditopic 3-pyridyl ligands is described.

## Introduction

Coordination-based self-assembly is underpinned by a set of well-established design principles. These geometrical tenets have enabled chemists to rationally target molecular architectures possessing an enormous range of structural and functional characteristics.^1,2^ The directional bonding approach is perhaps the most intuitive design strategy.^1g,3^ This approach entails the combination of donor (ligand) and acceptor (metal complex) units, the geometries of which determine the structure of the assembly formed. A fundamental requirement of this strategy is that these building blocks are shape-persistent; possessing conformational rigidity and well-defined coordinate vectors. The self-assembly behavior of building blocks without these characteristics is less predictable and, consequently, their strategic incorporation into supramolecular structures inherently difficult.

**Scheme 1:**
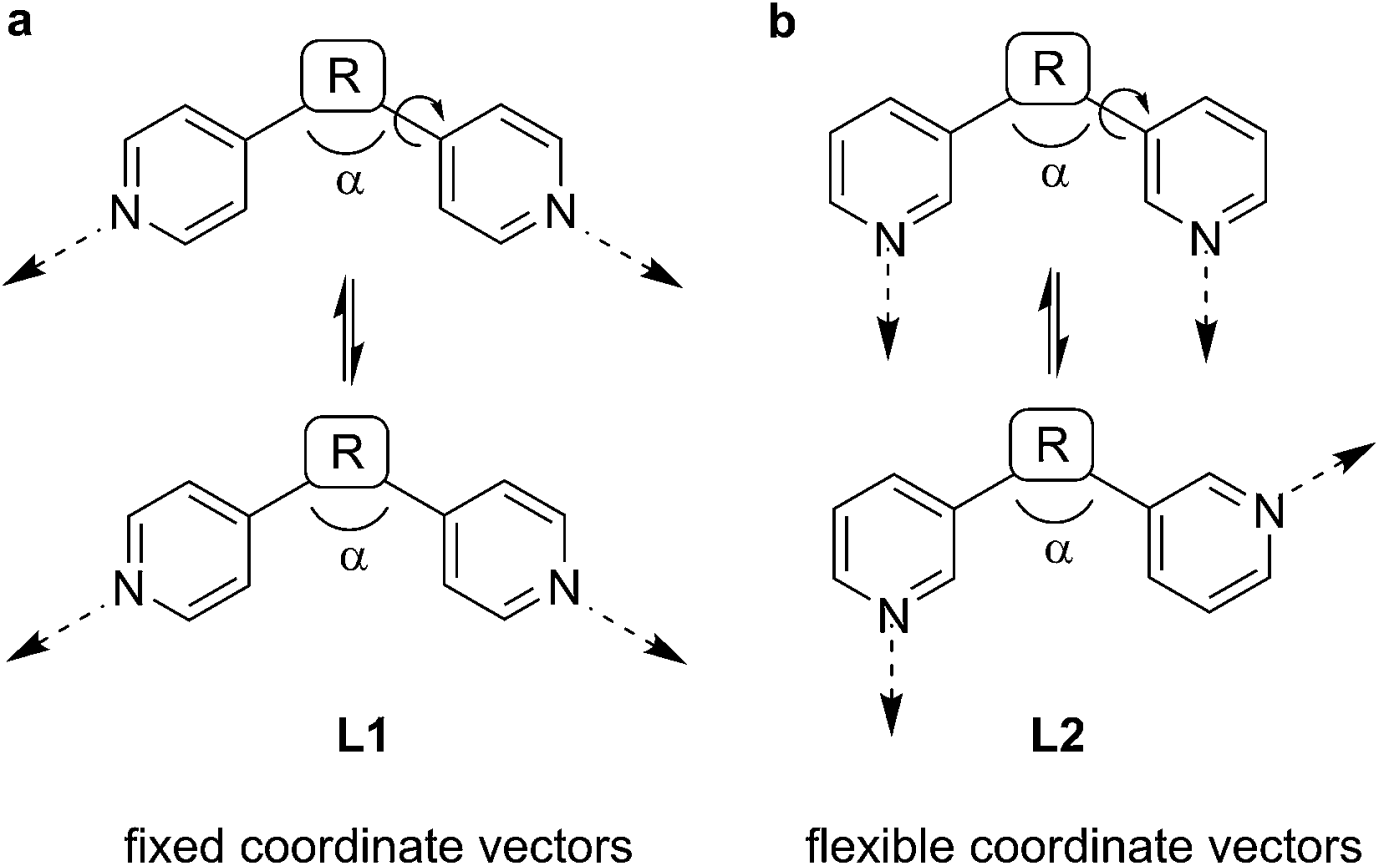
For ditopic ligands **L2** with 3-pyridyl donor groups, the relative orientation of the coordinate vectors is more flexible than for ligands **L1** with 4-pyridyl donor groups.

Ditopic N-donor ligands with terminal 4-pyridyl groups are amongst the most extensively exploited building blocks in the preparation of coordination-based supramolecular assemblies. The simplest member of this family of tectons is 4,4′-bipyridine, which has been incorporated into countless discrete^1^ and polymeric self-assembled structures.^3^ The insertion of a linker group between the 4-pyridyl moieties has led to ever more elaborate assemblies.^4,5^ In contrast to the ubiquity of 4-pyridyl terminated N-donor ligands, closely related 3-pyridyl terminated ligands are far less common.^1–3^ Scheme 1 alludes to why this is the case. The coordinate vectors of a generic ditopic N-donor ligand **L1** with terminal 4-pyridyl groups are fixed, regardless of the linking group R between the heterocycles, provided that R is rigid. Free rotation about the R–pyridine bond does not affect the orientation of the non-bonding sp^2^ orbitals of the N atoms (Scheme 1a). However, in the case of a generic 3-pyridyl terminated ligand **L2**, rotation about this bond changes the coordinate vectors of the pyridine N atoms (Scheme 1b).

**Scheme 2:**
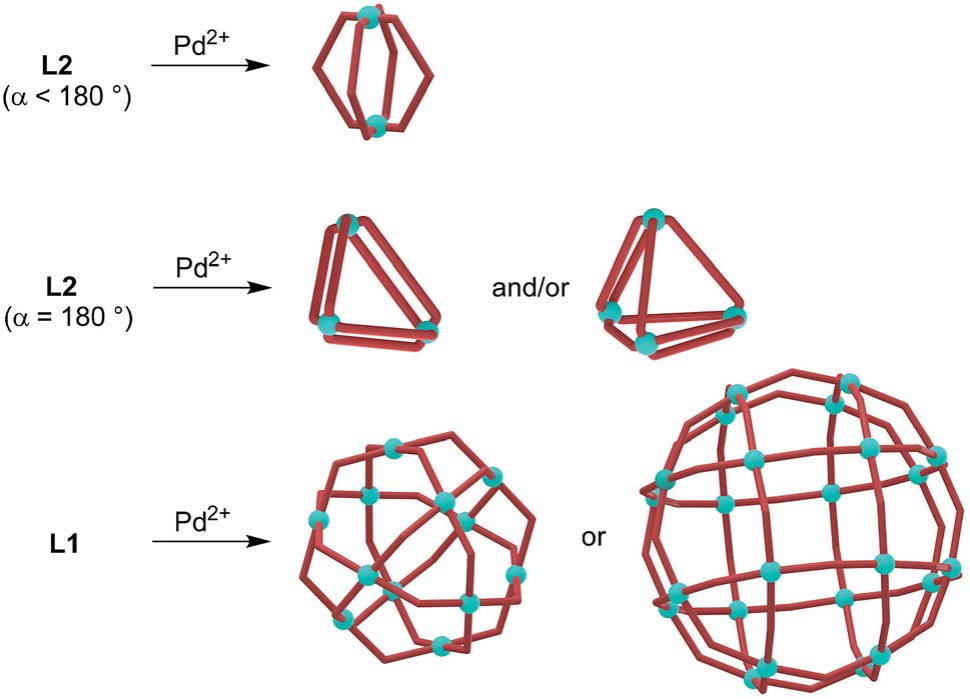
Reactions of Pd^2+^ with ligands of type **L2** give di-, tri- and tetranuclear aggregates, whereas large coordination cages are obtained upon reaction with ligands of type **L1**.

As a consequence of the conformational flexibility of 3-pyridyl terminated N-donor ligands, entropically favored small^6^ aggregates are obtained upon coordination to metal ions. Several research groups have shown that the combination of Pd^2+^ with ‘banana-shaped’ ligands of type **L2** (*α* < 180°) typically results in the formation of dinuclear aggregates of the formula [Pd_2_(**L2**)_4_]^4+^ (Scheme 2).^7,8^ Fujita and co-workers have examined the reaction of Pd^2+^ with linear ligands of type **L2** (*α* = 180°) and observed the formation of [Pd_3_(**L2**)_6_]^6+^ and [Pd_4_(**L2**)_8_]^8+^ complexes.^9^ These results are in contrast to reactions of Pd^2+^ with ligands of type **L1**, which were found to give polynuclear coordination cages of type [Pd_12_(**L1**)_24_]^24+^ and [Pd_24_(**L1**)_48_]^48+^.^4^ Inspection of the assemblies obtained with **L2**-type ligands reveals that they all feature Pd(**L2**)_2_Pd macrocycles as part of their structure.^10^ We hypothesized that increasing the steric bulk of **L2**-type ligands would overcome their entropic propensity to form small aggregates. Bulky lateral R group substituents should disfavor Pd(**L2**)_2_Pd macrocycles and thus result in more expanded assemblies. Below we show that this strategy can indeed be used to access unprecedented, large coordination cages based on ditopic 3-pyridyl ligands. In particular, we demonstrate that clathrochelate-based metalloligands enable the synthesis of octahedral [Pd_6_(**L2**)_12_]^12+^ cages with a diameter of more than 20 Å. Despite the lateral size of the metalloligands, the encapsulation of reasonably large guest molecules such as tetraphenylborate (BPh_4_^–^) is possible.

## Results and discussion

**Scheme 3:**
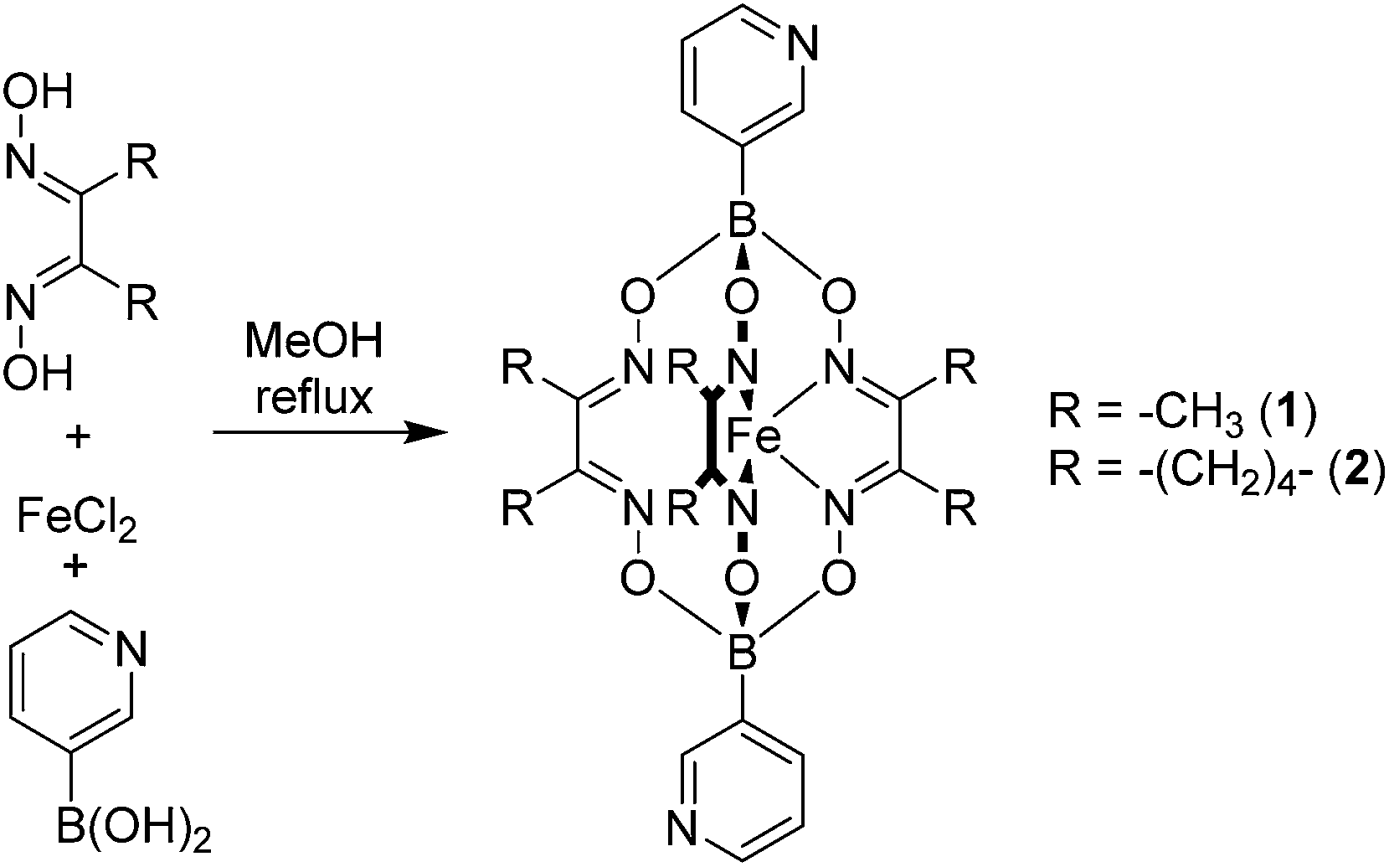
Synthesis of the bipyridyl ligands **1** and **2**.

Bipyridyl ligands **1** and **2** were isolated in 96% and 79% yield respectively, following a one-pot synthesis similar to that previously described for 4-pyridyl functionalized clathrochelates (Scheme 3).^11^ All the starting materials used in this procedure—1,2-cyclohexanedione dixoime (nioxime) or dimethylglyoxime, anhydrous iron(ii) chloride and pyridin-3-ylboronic acid—are commercially available.

**Fig. 1:**
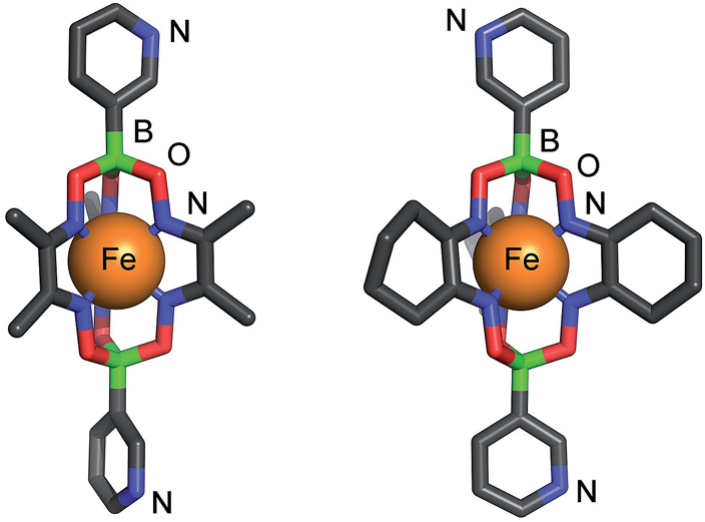
Molecular structures of clathrochelate-based bipyridyl ligands **1** (left) and **2** (right) as determined by X-ray crystallography. Color coding: C: gray, B: green, Fe: orange, N: blue, O: red. Hydrogen atoms and solvent molecules are omitted for clarity.

Single crystal X-ray diffraction unambiguously confirmed the formation of **1** and **2** (Fig. 1). The crystal structures reveal the conformational flexibility of the coordination vectors of these 3-pyridyl appended ligands. In **1**, the two planes of the pyridine rings are close to orthogonal (85.8°). Conversely, in **2**, they are almost coplanar (7.8°), yet the nonbonding sp^2^ orbitals of the pyridine N atoms are essentially parallel and divergent. The bond lengths and angles found for the trigonal prismatic Fe core of **1** and **2** are within the range observed for other Fe-based clathrochelate complexes.^12^

The inherent conformational flexibility of **1** and **2** makes the prediction of the structure of an assembly comprising these building blocks difficult, even when combined with a metal acceptor of well-defined geometry, such as a naked Pd^2+^ ion (square planar geometry). As outlined above, we envisaged that the considerable lateral steric bulk of **1** and **2** would play a structure-directing role and prevent the formation of macrocyclic Pd(**L2**)_2_Pd motifs. Consequently, we anticipated that larger cage structures could be formed by combination of **1** or **2** and a naked Pd^2+^ ion, rather than small aggregates.

**Scheme 4:**
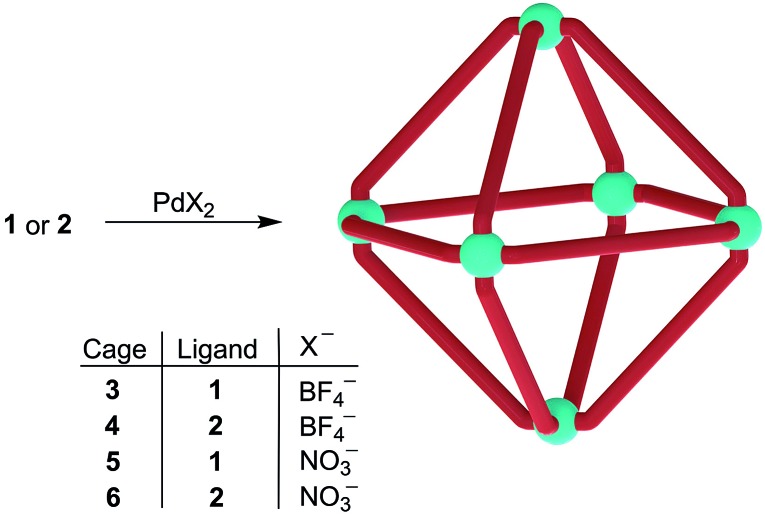
Synthesis of the cages **3–6**.

NMR-scale experiments were subsequently undertaken to investigate this hypothesis. A solution of [Pd(CH_3_CN)_4_](BF_4_)_2_ in acetonitrile-*d*_3_ was added to two equivalents of solid **1** or **2**, both of which are poorly soluble in acetonitrile-*d*_3_ at room temperature, giving a pale yellow suspension. These samples were then heated at 70 °C and ^1^H NMR spectra were recorded periodically. Over time, the reaction mixtures progressively became less turbid and turned red. After 3 hours, neither solution contained any precipitate and a single set of sharp peaks was observed in the ^1^H NMR spectrum in both cases, suggesting the formation of a single highly symmetric, discrete species (ESI, Fig. S19 and S20†). The syntheses were subsequently performed on a preparative scale, without deuterated solvent (Scheme 4). The ESI-MS spectra of the products show major peaks which can be attributed to complexes of the formula (Pd_6_(**L2**)_12_) (BF_4_)_*n*_^12–*n*+^ (*n* = 4–6, **L2** = **1** or **2**, ESI Fig. S1 and S2†). This result confirmed that higher order assemblies (**3**, **4**) had indeed been formed from **1** and **2** respectively, rather than small aggregates. The complexes **5** and **6** were prepared in an analogous fashion by heating a suspension of **1** or **2** and Pd(NO_3_)_2_(H_2_O)_*n*_ in a 5 : 1 mixture of acetonitrile : water for 2 hours at 70 °C. The ^1^H NMR spectra once more showed a single set of signals for each product and ESI-MS confirmed the molecular formulas of the cations to be [(Pd_6_(**1**)_12_)]^12+^ and [(Pd_6_(**2**)_12_)]^12+^ (ESI, Fig. S3 and S4†). Isolation of all complexes was achieved by precipitation with diethyl ether (isolated yields: 82–94%).

Single crystals of **3** suitable for X-ray diffraction were grown by diffusion of diethylether into a solution of the assembly in acetonitrile, whilst those of **5** and **6** were grown by layering an acetonitrile solution onto toluene. Synchrotron radiation was required to obtain diffraction data of sufficient quality, which were collected at the Swiss Norwegian Beamline at ESRF Grenoble. Assemblies of this size pose a number of difficulties due in part to their extensive inherent disorder and high solvent content within the crystal. Synchrotron radiation mitigates some of these difficulties, but we have also employed a series of carefully and rigorously adapted macromolecular refinement techniques^13^ in order to build a molecular model (see ESI† for details).

**Fig. 2:**
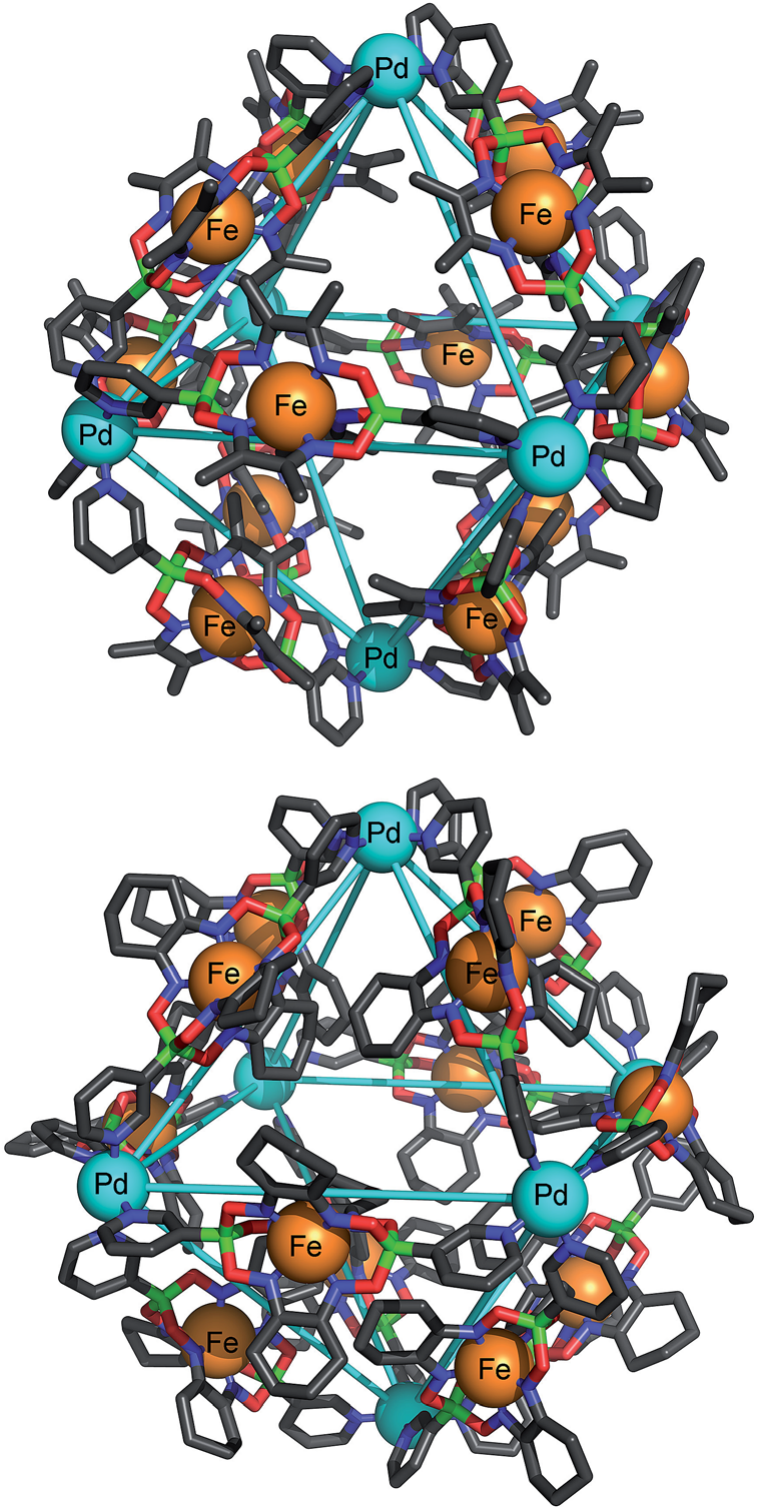
Molecular structure of cages **5** (top) and **6** (bottom) determined by X-ray crystallography. Color coding: C: gray, B: green, Fe: orange, N: blue, O: red, Pd: cyan. Anions and hydrogen atoms are omitted for clarity.

Fig. 2 shows the molecular structures of **5** and **6** in the crystal (the structure of **3** is similar to that of **5** and thus not shown). In all structures, the six Pd^2+^ ions of the assembly form an octahedron of close to regular geometry, with Pd–Pd distances of 2.19–2.32 nm across the body of the framework, and 1.52–1.63 nm within each triangular face. The internal angles of the facial triangles vary from 57.3° to 61.4°. The structural regularity of the Pd framework is not, however, reflected in the conformation of the bridging ligands themselves. In **5**, the planes of the pyridine rings of the six ligands along the edges of two opposing faces of the octahedral framework deviate significantly from parallel (plane dihedral angles 90.0°, 89.2° and 66.8°). In contrast, the planes of the pyridine rings of the remaining six ligands are much closer to parallel (20.6°, 9.3° and 13.7°). The situation is yet more disordered in the cases of **3** and **6**, where a range of dihedral angles between pyridine rings within each clathrochelate is observed (10.8–89.9°). This illustrates the intrinsic conformational flexibility of ligands of the type **L2**. Their variable coordination vectors determine the most energetically favorable conformation of the assembly as a whole, and enable this arrangement to be attained.

However, the different conformations adopted by **1** and **2** in the self-assembled architectures **3–6** are not manifested in the solution phase NMR spectra at room temperature. Only one set of peaks is visible, implying that the pyridyl rings of the ligands **1** and **2**, as well as the tris(dioxime) clathrochelate core, undergo rapid conformational changes (on the NMR timescale). The ^1^H NMR spectra of **3** and **4** were also recorded at 233 K (CD_3_CN). Significant line broadening was observed in all cases (ESI, Fig. S23 and S24†). The extent of line broadening was far greater in the case of **4** than **3**. This result is unsurprising, given the bulky cyclohexyl substituents of ligand **2** impose a higher steric barrier to rotation than the relatively small methyl substituents of ligand **1**.

The octahedral cages possess internal void space, which is largely occupied by solvent molecules and anions (both ordered and disordered) in the crystal. The X-ray structures of **3**, **5** and **6** show NO_3_^–^ or BF_4_^–^ anions in interior binding pockets adjacent to the Pd^2+^ ions at the vertices of the octahedral assemblies. The presence of close anion–Pd contacts is in line with what has been observed for many cages of type [Pd_2_(**L2**)_4_]^4+^.^7^ Simple geometric approximations reveal the volume of the octahedra described by the six Pd^2+^ ions in **3**, **5** and **6** to be ∼5.2 nm^3^. However, much of the interior volume of the assembly is occupied by clathrochelate oxime substituents.^14^ The influence of the lateral size of the clathrochelate complexes over the void volume within each assembly was quantified by VOIDOO calculations (see ESI† for details).^15^ In the solid state, assembly **3** possesses a cavity volume of 1.4 nm^3^ or 1.8 nm^3^ (two independent cages found in the asymmetric unit of the crystal structure), cage **5** a cavity volume of 1.3 nm^3^ and cage **6** a much smaller cavity volume of 1.1 nm^3^. From these values it is clear that it is not only the oxime substituents that significantly affect the internal space within each assembly, but also the conformation of the clathrochelate metalloligands. However, given the dynamic and fluxional nature of these cages evidenced by ^1^H NMR, this conformational influence was not expected to contribute to the solution phase void volume, and hence guest binding behavior, of assemblies **3–6**. Any differences in solution phase behavior can be attributed to the oxime substituents alone. To investigate whether oxime steric bulk would influence the kinetics and thermodynamics of guest binding, ^19^F NMR spectra of **3** and **4** were recorded at 298 K in acetonitrile-*d*_3_. A single peak was observed at –150.55 ppm for the BF_4_^–^ anion in **3**, whereas peaks at –146.37 ppm and –151.98 ppm were observed in the case of **4** (referenced to hexafluorobenzene at –164.90 ppm). These data suggest that the rate of exchange between internal and external BF_4_^–^ is fast on the NMR timescale at 298 K in the case of **3** (one signal), but slow in the case of **4** (two signals). ^19^F NMR spectra of **3** and **4** were subsequently recorded over a range of temperatures (see ESI, Fig. S25 and S26†). These experiments revealed a coalescence temperature of approximately 255 K for the BF_4_^–^ anion signals of **3**, and 345 K for those of **4**. The difference of 90 K corresponds to a difference of ∼16 kJ mol^–1^ for the activation free energy^16^ of the anion exchange process and neatly illustrates one key characteristic of clathrochelate-based building blocks in general—the ease with which we can modulate their steric bulk.

In addition to the anion binding sites situated adjacent to the Pd^2+^ metal centers, cages **3–6** possess a central cavity. The internal environment of this cavity is hydrophobic due to the methyl or cyclohexyl substituents of the clathrochelate ligands, yet the overall charge of the cages is 12+. Consequently, we expected coulombic interactions and the hydrophobic effect to dominate the guest binding behavior of assemblies **3–6**. Therefore, the lipophilic tetraphenylborate (BPh_4_^–^) anion appeared to be a potentially suitable guest molecule. The BPh_4_^–^ anion was of particular interest not only given its perceived size and electrostatic match, but also the limited number of host complexes that have been found to bind BPh_4_^–^ as a guest. To the best of our knowledge, only certain cyclodextrins,^17^ cavitands,^18^ liposomes^19^ and the external face of a metallosupramolecular cube^20^ have been observed to associate with BPh_4_^–^ in a host—guest manner.

Complexation studies with cage **4** were hampered by the formation of precipitates upon addition of NaBPh_4_, and ultimately abandoned. However, addition of one equivalent of NaBPh_4_ to **3** (0.62 mM) in CD_3_CN did not lead to precipitation, and significant changes in the ^1^H NMR signals of both species were apparent.^21^ Two sets of peaks were immediately visible for the protons of the phenyl groups of the anionic guest, and a new set of signals was also observed for aromatic protons and methyl groups of the clathrochelate ligands. These observations were consistent with the formation of a host—guest complex between the cage and the BPh_4_^–^ anion, the rate of exchange of bound and unbound BPh_4_^–^ being slow on the NMR timescale. To confirm the encapsulation of the BPh_4_^–^ anion within the interior cavity of **3**, DOSY and NOESY NMR experiments were performed. DOSY NMR revealed a common diffusion coefficient (4.94 × 10^–6^ cm^2^ s^–1^) for the assembly and one of the two sets of BPh_4_^–^ protons (ESI, Fig. S40–S43†), whilst NOESY cross peaks were observed between the CH_3_ groups of the clathrochelate oxime ligand and the same set of BPh_4_^–^ peaks (ESI, Fig. S46–S49†).

The apparent association constant for the complexation of BPh_4_^–^ by **3** was calculated through integration of the NMR signals corresponding to bound and unbound guest across a range of concentrations, using a 1 : 1 binding model (see ESI† for details). The resulting value is *K*_a_ = 2.4(±0.3) × 10^3^ M^–1^. We subsequently recorded the ^1^H NMR spectra of **3** in the presence of one equivalent of NaBPh_4_ in a 2 : 1 mixture of CD_3_CN and D_2_O, and a 1 : 1 mixture of CD_3_CN and CDCl_3_ in order to elucidate the effect of solvent polarity upon BPh_4_^–^ binding. In 2 : 1 CD_3_CN : D_2_O, the binding constant was calculated to be of the order of 10^5^ M^–1^, whereas in 1 : 1 CD_3_CN : CDCl_3_, the intensities of the peaks corresponding to bound BPh_4_^–^ were too low to integrate reliably. The large increase in binding affinity observed upon changing the solvent from neat CD_3_CN to the more polar 2 : 1 CD_3_CN : D_2_O mixture, as well as the dramatic drop observed in the much less polar 1 : 1 CD_3_CN : CDCl_3_ mixture, enforces the hypothesis that the hydrophobic effect plays a significant role in guest binding. These apparent association constants take into account not only the encapsulation of BPh_4_^–^ by **3**, but also the thermodynamically unfavorable concomitant ejection of internal BF_4_^–^. Consequently, the affinity of **3** for BPh_4_^–^ is, in isolation, presumably greater than the composite association constant recorded as discussed above.

**Fig. 3:**
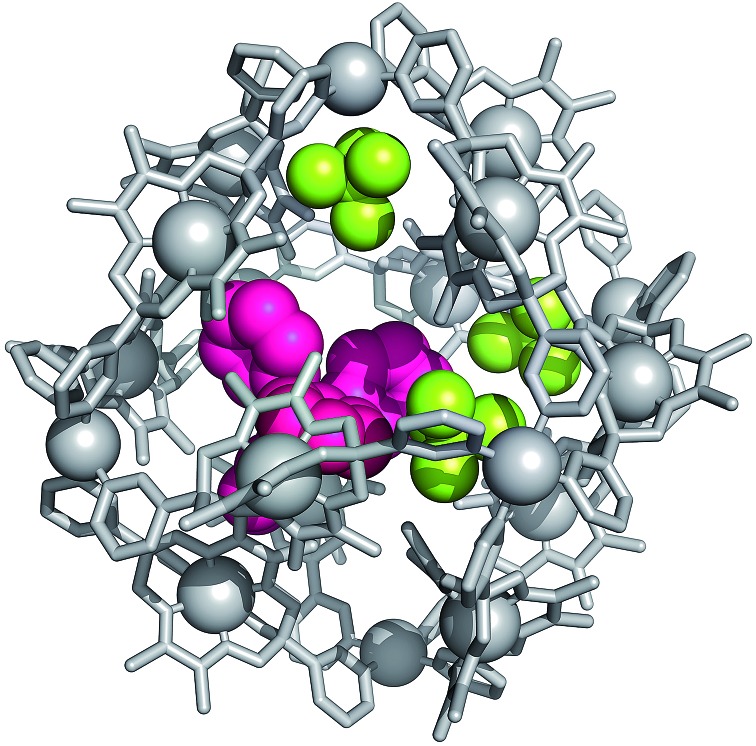
Part of the X-ray crystal structure of **7**, highlighting the encapsulated BF_4_^–^ (yellow-green) and BPh_4_^–^ (magenta) anions. External anions, hydrogen atoms and solvent molecules are omitted for clarity.

Encapsulation of BPh_4_^–^ by **3** was unambiguously confirmed by single crystal X-ray crystallography. A ten-fold excess of NaBPh_4_ was added to a solution of **3** in 2 : 1 CD_3_CN : D_2_O, and from the resulting suspension single crystals of a mixed salt (**7**) containing both BPh_4_^–^ and BF_4_^–^ anions were isolated. Despite the relatively poor quality of the diffraction data, due to the size and complexity of the structure, a sufficient solution was obtained, which unquestionably established the connectivity within the [Pd_6_ **1**_12_] assembly, as well as the presence of encapsulated BPh_4_^–^. Unsurprisingly, it was impossible to locate all BF_4_^–^ and BPh_4_^–^ anions due to the extent to which they are disordered, hence precise formulation of this salt cannot be undertaken using the X-ray diffraction data. Rather, the general formula [BPh_4_@Pd_6_ **1**_12_][BF_4_]_*m*_[BPh_4_]_*n*_, where *m* + *n* = 11, must be used to describe **7**. Two independent cages were found in the asymmetric unit, each of which contained a single encapsulated BPh_4_^–^ (see ESI, Fig. S31†). One of the two independent cages additionally contained three ordered BF_4_^–^ (Fig. 3), whilst a single ordered BF_4_^–^ was located in the second. The positions of the different anions in the former illustrate the differences in size of the two interior cavities of **3**—the smaller BF_4_^–^ anions occupy binding pockets adjacent to the Pd^2+^ vertices of one face of the octahedral framework, whilst the non-coordinating BPh_4_^–^ resides within the larger central cavity of the assembly. Two of the phenyl groups of BPh_4_^–^ sit snugly between the methyl substituents of the clathrochelate oxime ligands of two neighboring faces of the [Pd_6_ **1**_12_]^12+^ assembly. This conformation alludes to the contribution of hydrophobic interactions to BPh_4_^–^ encapsulation, and is consistent with the upfield change in chemical shift observed for the protons of the CH_3_ groups of the oxime substituents as they are exposed to the diamagnetic ring current of the phenyl rings (see ESI, Fig. S37†).

## Conclusions

Herein, we have reported the syntheses and the structures of two clathrochelate-based metalloligands with terminal 3-pyridyl groups (**1** and **2**). Reactions of these ligands with Pd^2+^ afford large octahedral coordination cages (**3–6**), which were comprehensively characterized (including crystallographic analysis for three of the four cages). The formation of octahedral Pd cages from ditopic ligands containing 3-pyridyl groups is unprecedented. Typically, conformationally flexible ligands with terminal 3-pyridyl groups give rise to small aggregates (Scheme 2). In our case, the lateral size of the metalloligands prevents the formation of macrocyclic Pd(**L2**)_2_Pd structures. This enthalpic effect (steric hindrance) is sufficient to overcome the entropically driven propensity of a coordination-based supramolecular system to form small aggregates. Consequently, larger octahedral structures are obtained, the assembly of which is favored to such an extent that no side products are observed. Despite the fact that we have used steric effects to enlarge the assembly, we were still able to obtain cages with rather large cavities. Notably, cage **3** was found to bind the ‘non-coordinating’^22^ anion BPh_4_^–^ with a solvent-dependent association constant of the order of 10^5^ M^–1^.

Our work also highlights the advantageous characteristics of clathrochelate complexes as building blocks for supramolecular chemistry.^11,23^ These complexes are straightforward to synthesize and their lateral size and functionality^24^ can be modulated by the boronic acid capping groups and the oxime substituents. The ability to modify the structure of the metalloligands without substantial synthetic efforts represents an important advantage for implementing or optimizing a certain function of the final assembly. As a proof-of-principle study, we have shown that the kinetics of BF_4_^–^ exchange are strongly affected by the nature of the oxime-derived side chain (methyl *vs.* cyclohexyl). We believe that judicious design of clathrochelate building blocks will enable many more structurally and functionally novel self-assembled architectures to be obtained, and we are continuing to pursue the applications of these ligands in coordination-based self-assembly.
