# Supplementary material for: Large, heterometallic coordination cages based on ditopic metallo-ligands with 3-pyridyl donor groups
Source: Chem Sci. 2014 Nov 11;6(2):1004–10. doi: 10.1039/c4sc03046j (PMC5811078; doi:10.1039/c4sc03046j)
Supplement: Supplementary file 1 [file SC-006-C4SC03046J-s001.pdf]

Electronic Supplementary Material (ESI) for Chemical Science.
This journal is © The Royal Society of Chemistry 2014

Electronic Supplementary Information

Large, heterometallic coordination cages based on ditopic metalloligands with 3-pyridyl donor groups

Matthew D. Wise,[a] Julian J. Holstein,[b] Philip Pattison,[c,d] Celine Besnard,[e] Euro Solari,[a] Rosario Scopelliti,[a] Gerard Bricogne[f] and Kay Severin[a,]*

a) Institut des Sciences et Ingénierie Chimiques, École Polytechnique Fédérale de Lausanne, CH-1015 Lausanne, Switzerland

b) GZG, Abteilung Kristallographie, Georg-August-Universität Göttingen, Goldschmidtstr. 1, 37077 Göttingen, Germany

c) Swiss-Norwegian Beamline, ESRF, F-Grenoble

d) Laboratory of Crystallography, École Polytechnique Fédérale de Lausanne, CH-1015 Lausanne, Switzerland

e) Laboratory of X-ray Crystallography, University of Geneva, CH-1211-Geneva 4, Switzerland

f) Global Phasing Ltd., Sheraton House, Castle Park Cambridge CB3 0AX, England

Table of Contents

1. General S3

2. Synthetic Procedures S4

3. Mass Spectrometric Analysis S7

4. NMR Spectra S9

  4.1 Routine ^{1}H, ^{13}C and ^{19}F Spectra of 1–6 S9

  4.2 Monitoring the Formation of 3–6 S16

  4.3 Low Temperature ^{1}H NMR Spectra of 3 and 4 S19

  4.4 Variable Temperature ^{19}F NMR Spectra of 3 and 4 S20

5. Single Crystal X-ray Analyses S22

  5.1 General S22

  5.2 Ligands 1 and 2 S23

  5.3 Assemblies **3**, **5**, **7** and **6** S25

  5.3 Cavity Volume Calculations S32

6. Encapsulation of the BPh_4 Anion S35

  6.1 Experimental Methodology S35

  6.2 DOSY NMR Spectra S37

  6.3 NOESY NMR Spectra S39

  6.4 Calculation of Association Constants S43

7. References S45

1. General

All chemicals were obtained from commercial sources and used without further purification. $Pd(NO_3)_2(H_2O)_n$ was supplied as 40% Pd by weight. Solvents were dried using a solvent purification system from Innovative Technologies, Inc.. Reactions were carried out under an atmosphere of dry N_2 using standard Schlenk techniques. Routine ^1H, ^{19}F and ^{13}C NMR spectra were obtained on a Bruker Avance III spectrometer (^1H: 400 MHz, ^{19}F: 376 MHz, ^{13}C: 101 MHz) equipped with a 5 mm BBFO-Plus$_z$ probe. Low and elevated temperature ^{19}F spectra were obtained on a Bruker DRX spectrometer (^1H: 400 MHz, ^{19}F: 376 MHz) equipped with a 5 mm BBO probe, and DOSY and NOESY NMR spectra were recorded on a Bruker Avance spectrometer (^1H: 400 MHz) equipped with a 5 mm BBIz probe. ^1H chemical shifts are reported in parts per million δ (ppm) referenced to an internal solvent. ^{19}F chemical shifts are reported in ppm and referenced to external hexafluorobenzene in CD_3CN (−164.90 ppm). ^{13}C chemical shifts are reported in ppm and referenced to an internal solvent. All routine, and DOSY and NOESY NMR spectra were recorded at 298K. Electrospray-ionization MS data were acquired on a Q-TOF Ultima mass spectrometer (Waters) operated in the positive ionization mode and fitted with a standard Z-spray ion source equipped with the Lock-Spray interface. Data were processed using the MassLynx 4.1 software.

2. Synthetic Procedures

Ligand 1

Dimethylglyoxime (708 mg, 6.10 mmol), pyridin-3-ylboronic acid (500 mg, 4.07 mmol) and anhydrous $FeCl_2$ (257 mg, 2.03 mmol) were suspended in MeOH (40 mL) and heated under reflux for 3 h under N_2 to give a deep red solution. This reaction mixture was allowed to cool to RT before the volume of solvent was reduced by half under vacuum. An orange precipitate formed upon the addition of Et_2O (50 mL). This solid was collected by centrifugation before being dissolved in $CHCl_3$, transferred to a separatory funnel, washed with saturated aqueous $NaHCO_3$ (3 x 100 mL) and water (100 mL), dried ($MgSO_4$), and filtered before solvent was removed under vacuum to give **1** as a red solid (1.12 g, 96%); ^{1}H NMR (400 MHz, CD_2Cl_2) δ = 8.85 (s, 2H), 8.53 (d, J = 3.4 Hz, 2H), 7.98 (d, J = 7.4 Hz, 2H), 7.27 (dd, J = 7.4, 4.9 Hz, 2H), 2.42 (s, 18H); ^{13}C NMR (101 MHz, CD_2Cl_2) δ = 153.43, 153.17, 149.40, 139.84, 123.41, 13.74 (C-B not detected); HRMS (ESI): *m/z* calculated for $C_{22}H_{26}B_2FeN_8O_6$ $[M+2H]^{++}$ 289.0838, found 289.0825. X-ray quality single crystals of **1** were obtained by slow evaporation of a solution of the compound in $CDCl_3$.

Ligand 2

Nioxime (1.73 g, 12.2 mmol), pyridin-3-ylboronic acid (1.00 g, 8.14 mmol) and anhydrous $FeCl_2$ (514 mg, 4.06 mmol) were dissolved in MeOH (80 mL) and heated under reflux for 4 h under N_2. The deep red reaction mixture was concentrated to dryness on a rotary evaporator before being redissolved in a minimum volume of CH_2Cl_2, transferred to a separatory funnel, and washed with saturated aqueous $NaHCO_3$ (3 x 100 mL) and water (100 mL), dried ($MgSO_4$), and filtered before solvent was removed under vacuum. The resulting red powder was recrystallized from MeOH to give **2** as a crystalline solid (2.10 g, 79%); ^{1}H NMR (400 MHz, CD_2Cl_2) δ = 8.80 (d, J = 1.7 Hz, 2H), 8.51 (dd, J = 4.9, 1.9 Hz, 2H), 7.93 (dt, J = 7.5, 1.8 Hz, 2H), 7.24 (dd, J = 7.5, 4.8 Hz, 2H), 2.92 (broad m, 12H), 1.82 (broad m, 12H); ^{13}C NMR (101 MHz, CD_2Cl_2) δ = 153.46, 152.89, 149.45, 139.81, 123.40, 26.80, 22.14 (C-B not detected); HRMS (ESI): *m/z* calculated for $C_{28}H_{34}B_2FeN_8O_6$ $[M+2H]^{++}$ 328.1074, found 328.1075. Single crystals of **2** suitable for X-ray diffraction were obtained from the preparative recrystallization from MeOH.

Cage 3

To ligand **1** (52 mg, 0.090 mmol) and $[Pd(MeCN)_4](BF_4)_2$ (20 mg, 0.045 mmol) in a Schlenk tube was added MeCN (6 mL) and $CHCl_3$ (3 mL), and the resulting suspension was heated to 70 ºC for 3 h under N_2. During this time, the initial suspension became a slightly turbid, deep red solution. After 3 h, the reaction mixture was cooled to RT, filtered and the volume of solvent was reduced by half under vacuum. Upon addition of Et_2O (10 mL) a precipitate formed. This orange solid was isolated by centrifugation and washed with pentane (10 mL) to give **3** (58 mg, 90%); ^1H NMR (400 MHz, CD_3CN) δ = 8.56 (s, 2H), 8.25 (d, J = 5.6 Hz, 2H), 8.20 (d, J = 7.9 Hz, 2H), 7.45 (dd, J = 7.6, 5.8 Hz, 2H), 2.23 (s, 18H); ^{13}C NMR (101 MHz, CD_3CN) δ = 155.51, 155.09, 151.93, 144.97, 127.18, 13.88 (C–B not detected); ^{19}F NMR (376 MHz, CD_3CN) δ = −150.55; ESI-MS: m/z calculated for $[M{-}6BF_4]^{6+}$ 1345.20, found 1345.22, $[M{-}7BF_4]^{7+}$ 1140.61 found 1140.62, $[M{-}8BF_4]^{8+}$ 987.16 found 987.16, $[M{-}9BF_4]^{9+}$ 867.80 found 867.82. Single crystals of **3** suitable for X-ray diffraction were grown by slow diffusion of diethylether into a solution of complex **3** in MeCN.

Cage 4

To ligand **2** (58 mg, 0.089 mmol) and $[Pd(MeCN)_4](BF_4)_2$ (20 mg, 0.045 mmol) in a Schlenk tube was added MeCN (9 mL), and the resulting suspension was heated to 70 ºC for 3 h under N_2. During this time, the suspension became a slightly turbid, deep red solution. After 3 h, the reaction mixture was cooled to RT, filtered and the volume of solvent was reduced by half under vacuum. Upon addition of Et_2O (10 mL) a precipitate formed. This orange solid was isolated by centrifugation and washed with pentane (10 mL) to give **4** (67 mg, 94%); ^1H NMR (400 MHz, CD_3CN) δ = 8.69 (s, 2H), 8.32 (d, J = 5.8 Hz, 2H), 8.13 (d, J = 7.2 Hz, 2H), 7.43 (dd, J = 7.6, 5.8 Hz, 2H), 2.73 (broad s, 12H), 1.69 (broad s, 12H); ^{13}C NMR (101 MHz, CD_3CN) δ = 155.65, 154.43, 151.66, 144.94, 127.11, 27.19, 22.09 (C-B not detected); ^{19}F NMR (376 MHz, CD_3CN) δ = −146.22, −152.07; ESI-MS: m/z calculated for $[M{-}5BF_4]^{5+}$ 1818.96, found 1818.99, $[M{-}6BF_4]^{6+}$ 1501.30 found 1501.44, $[M{-}7BF_4]^{7+}$ 1274.40 found 1274.38, $[M{-}8BF_4]^{8+}$ 1104.23 found 1104.11.

Cage 5

In a vial, **1** (44 mg, 0.076 mmol) was suspended in MeCN (5 mL) before $Pd(NO_3)_2(H_2O)_n$ (10 mg, 0.038 mmol) was added as a solution in H_2O (1 mL). The resulting suspension was

heated at 70 ºC for 2 h before being allowed to cool to RT. The slightly turbid deep red reaction mixture was filtered before Et_2O (10 mL) was added and an orange precipitate formed. This precipitate was collected by centrifugation and washed with pentane (10 mL) to give **5** (43 mg, 82%); ^{1}H NMR (400 MHz, CD_3CN) δ = 8.75 (s, 2H), 8.36 (dd, J = 6.0, 1.5 Hz, 2H), 8.16 (dt, J = 7.6, 1.5 Hz, 2H), 7.43 (dd, J = 7.8, 5.9 Hz, 2H), 2.25 (s, 18H); ^{13}C NMR (101 MHz, CD_3CN) δ = 155.90, 155.01, 151.88, 144.63, 126.86, 13.87 (C-B not detected); ESI-MS: m/z calculated for $[M{-}6NO_3]^{6+}$ 1320.19 found 1320.75, $[M{-}7NO_3]^{7+}$ 1122.74 found 1122.84, $[M{-}8NO_3]^{8+}$ 974.64 found 974.64, $[M{-}9NO_3]^{9+}$ 859.47 found 859.62. Single crystals of **5** suitable for X-ray diffraction were grown by layering a solution of complex **5** in MeCN onto toluene.

Cage 6

In a vial, **2** (50 mg, 0.076 mmol) was suspended in MeCN (5 mL) before $(Pd(NO_3)_2(H_2O)_n$ (10 mg, 0.038 mmol) was added as a solution in H_2O (1 mL). The resulting suspension was heated at 70 ºC for 2 h before being allowed to cool to RT. The slightly turbid deep red reaction mixture was filtered before Et_2O (10 mL) was added and an orange precipitate formed. This precipitate was collected by centrifugation and washed with pentane (10 mL) to give **5** (49 mg, 84%); ^{1}H NMR (400 MHz, 1:1 CD_3CN:D_2O) δ = 8.58 (s, 2H), 8.30 (d, J = 5.9 Hz, 2H), 8.07 (d, J = 7.5 Hz, 2H), 7.40 (apparent t, J = 6.8 Hz, 2H), 2.65 (broad s, 12H), 1.61 (broad s, 12H); ^{13}C NMR (101 MHz, CD_3CN) δ = 154.66, 153.95, 151.22, 144.30, 126.65, 26.58, 21.52 (C-B not detected); ESI-MS: m/z calculated for $[M{-}6NO_3]^{6+}$ 1476.29, found 1476.54, $[M{-}7NO_3]^{7+}$ 1256.53 found 1256.71, $[M{-}8NO_3]^{8+}$ 1091.72 found 1091.93, $[M{-}9NO_3]^{9+}$ 963.53 found 963.54. Single crystals of **6** suitable for X-ray diffraction were grown by layering a solution of complex **6** in MeCN onto toluene.

3. Mass Spectrometric Analysis

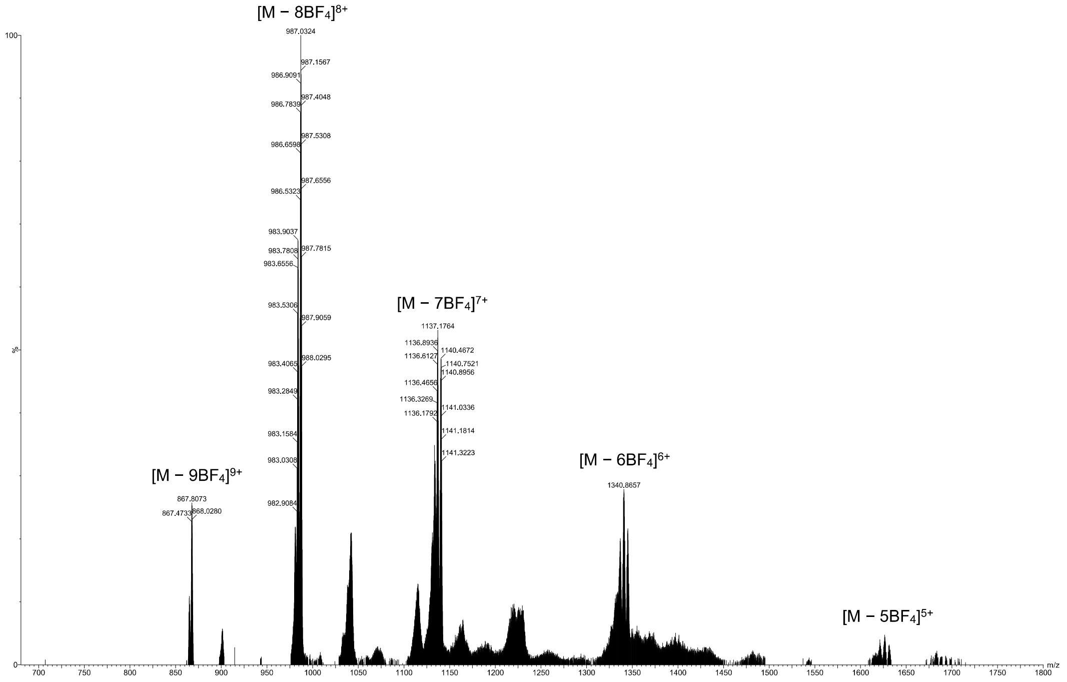

Figure S1. ESI MS spectrum of complex **3**. Peaks labeled correspond to $[M{-}5BF_4]^{5+}$, $[M{-}6BF_4]^{6+}$, $[M{-}7BF_4]^{7+}$, $[M{-}8BF_4]^{8+}$ and $[M{-}9BF_4]^{9+}$.

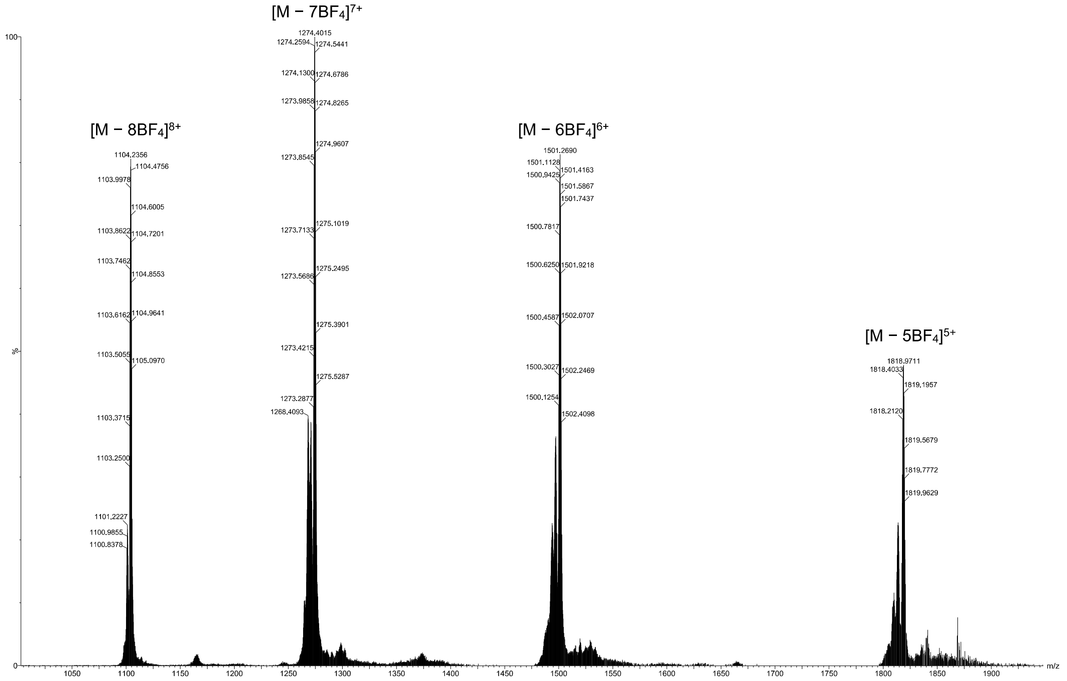

Figure S2. ESI MS spectrum of complex **4**. Peaks labeled correspond to $[M{-}5BF_4]^{5+}$, $[M{-}6BF_4]^{6+}$, $[M{-}7BF_4]^{7+}$ and $[M{-}8BF_4]^{8+}$.

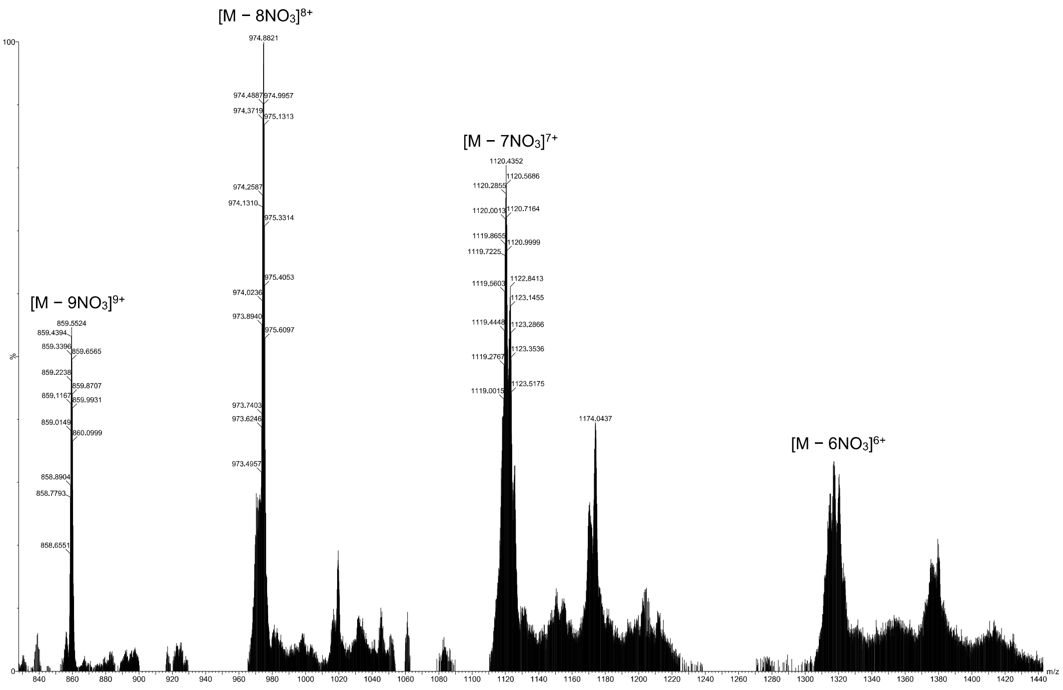

Figure S3. ESI MS spectrum of complex **5**. Peaks labeled correspond to $[M{-}6NO_3]^{6+}$, $[M{-}7NO_3]^{7+}$, $[M{-}8NO_3]^{8+}$ and $[M{-}9NO_3]^{9+}$.

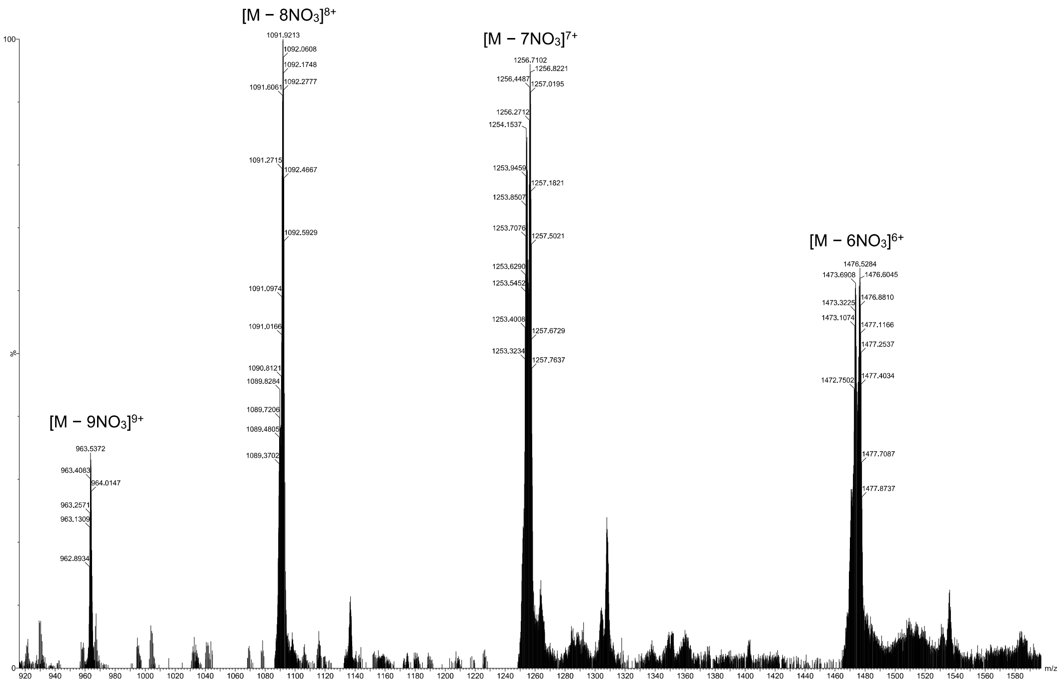

Figure S4. ESI MS spectrum of complex **6**. Peaks labeled correspond to $[M{-}6NO_3]^{6+}$, $[M{-}7NO_3]^{7+}$, $[M{-}8NO_3]^{8+}$ and $[M{-}9NO_3]^{9+}$.

4. NMR Spectra

4.1 Routine ^{1}H, ^{13}C and ^{19}F Spectra of 1–6

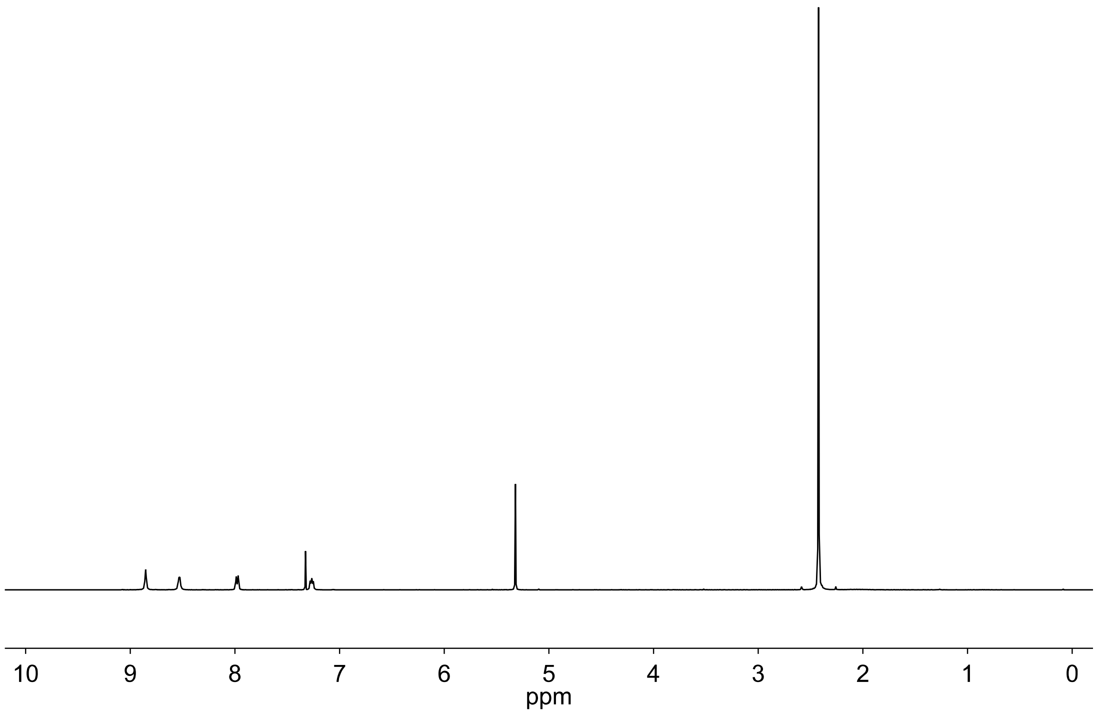

Figure S5. ^{1}H NMR spectrum of **1** recorded in CD_2Cl_2.

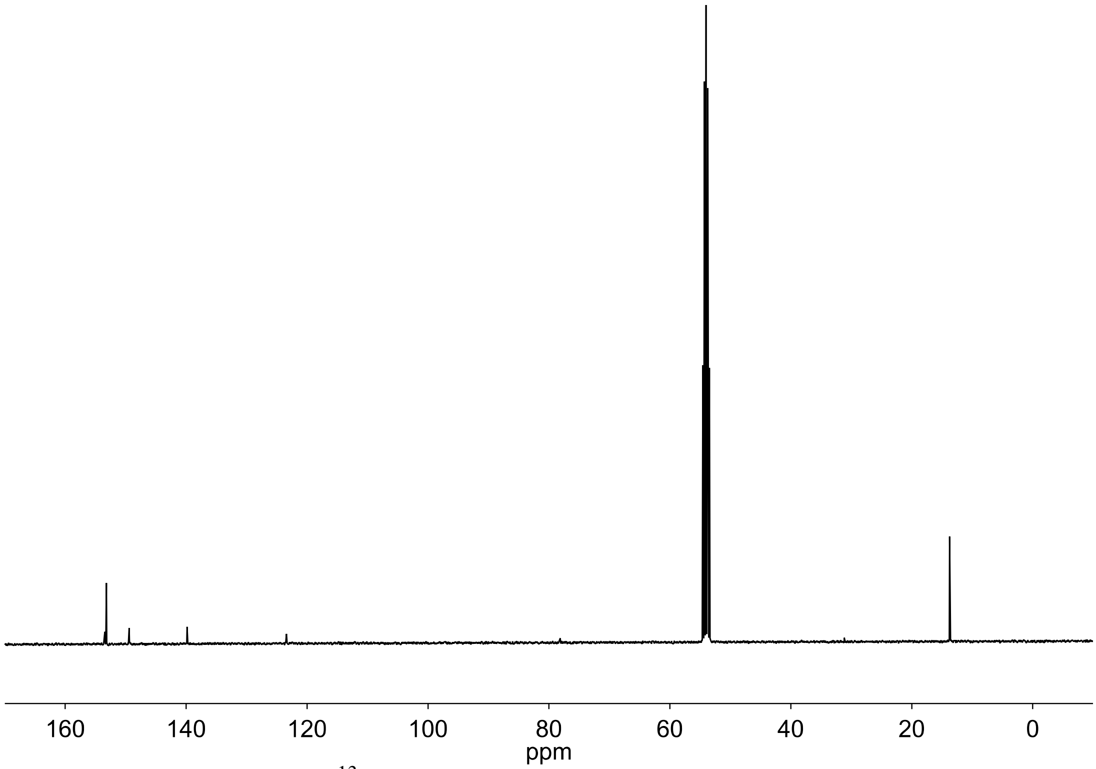

Figure S6. ^{13}C NMR spectrum of **1** recorded in CD_2Cl_2.

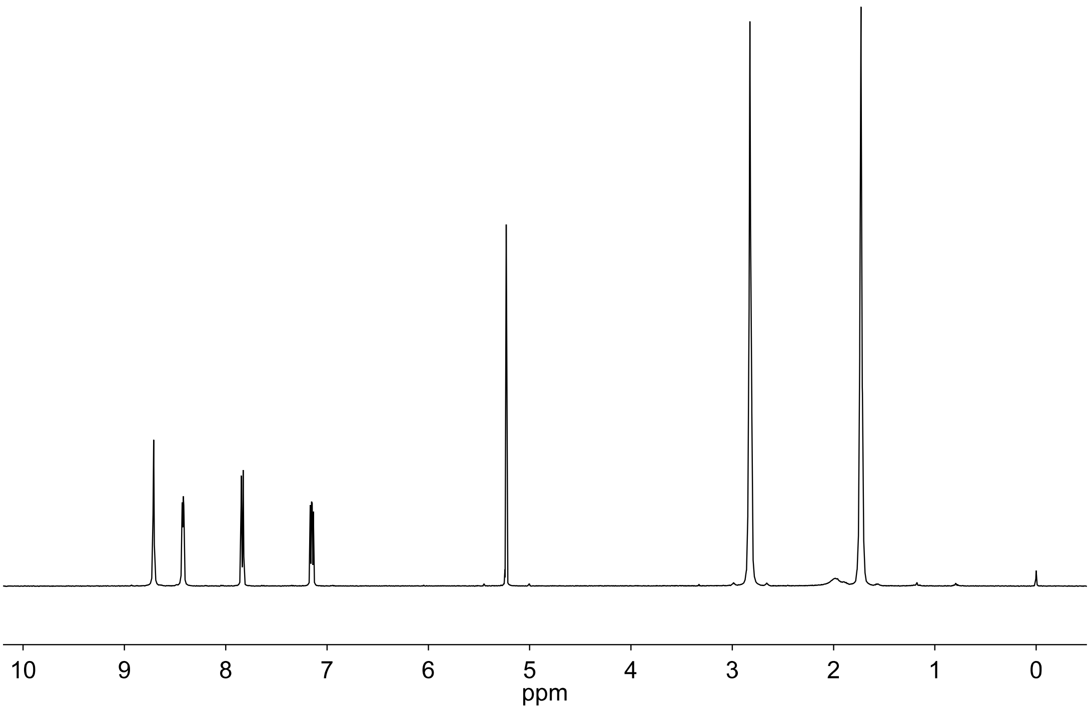

Figure S7. ^{1}H NMR spectrum of **2** recorded in CD_2Cl_2.

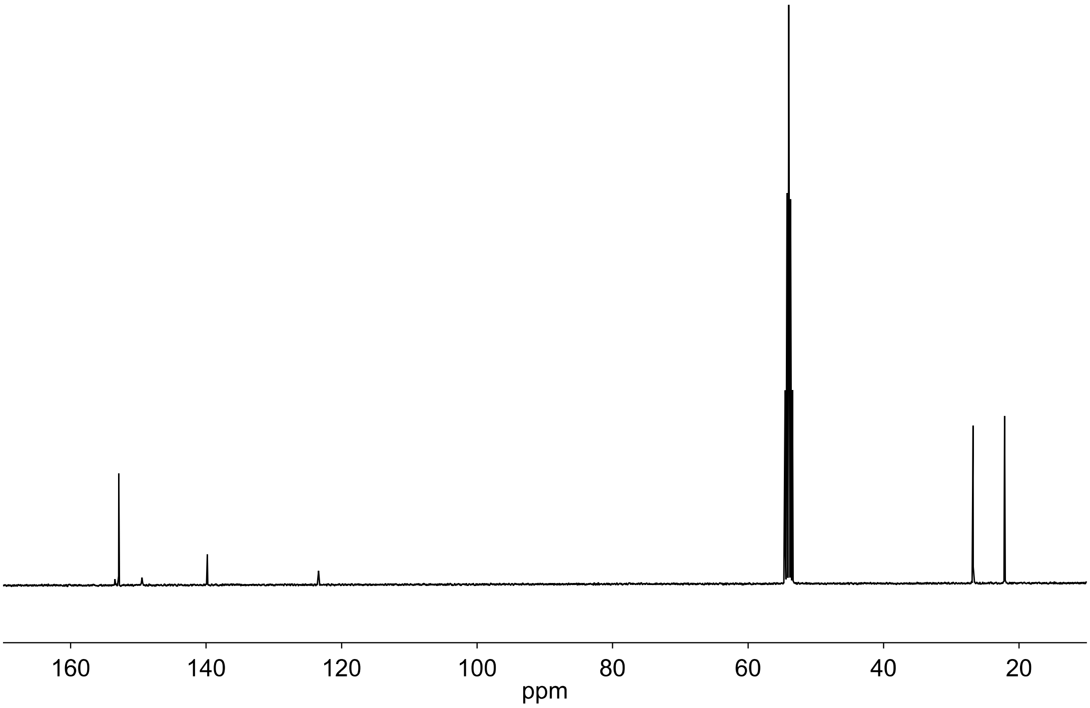

Figure S8. ^{13}C NMR spectrum of **2** recorded in CD_2Cl_2.

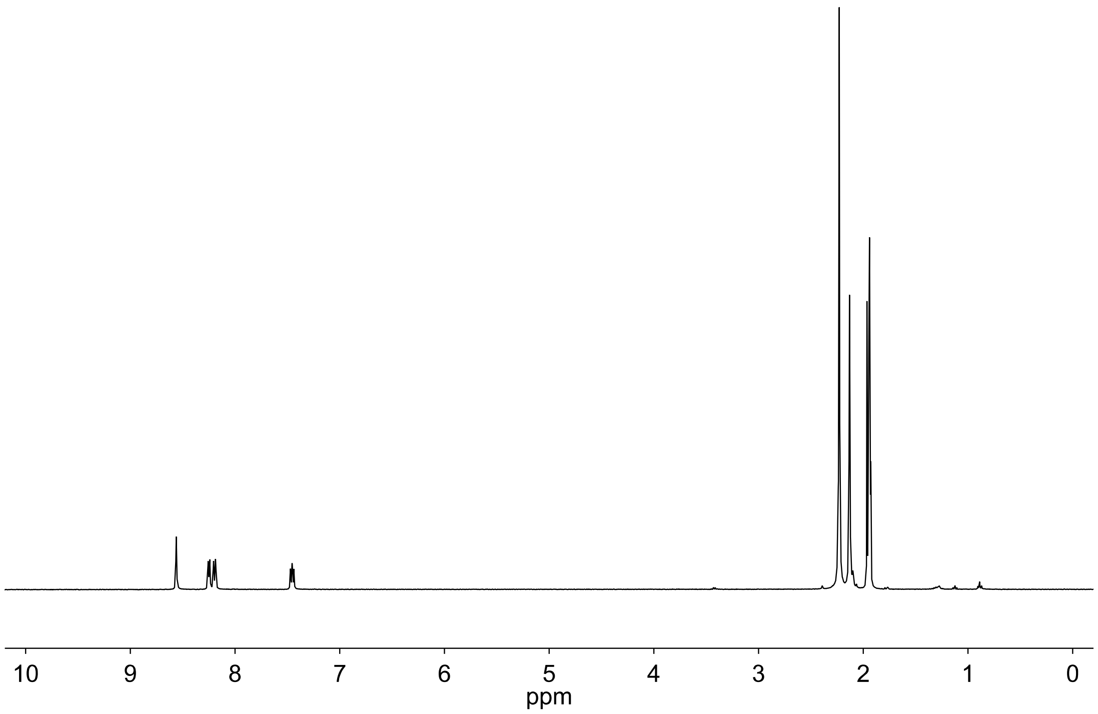

Figure S9. ^{1}H NMR spectrum of **3** recorded in CD_3CN.

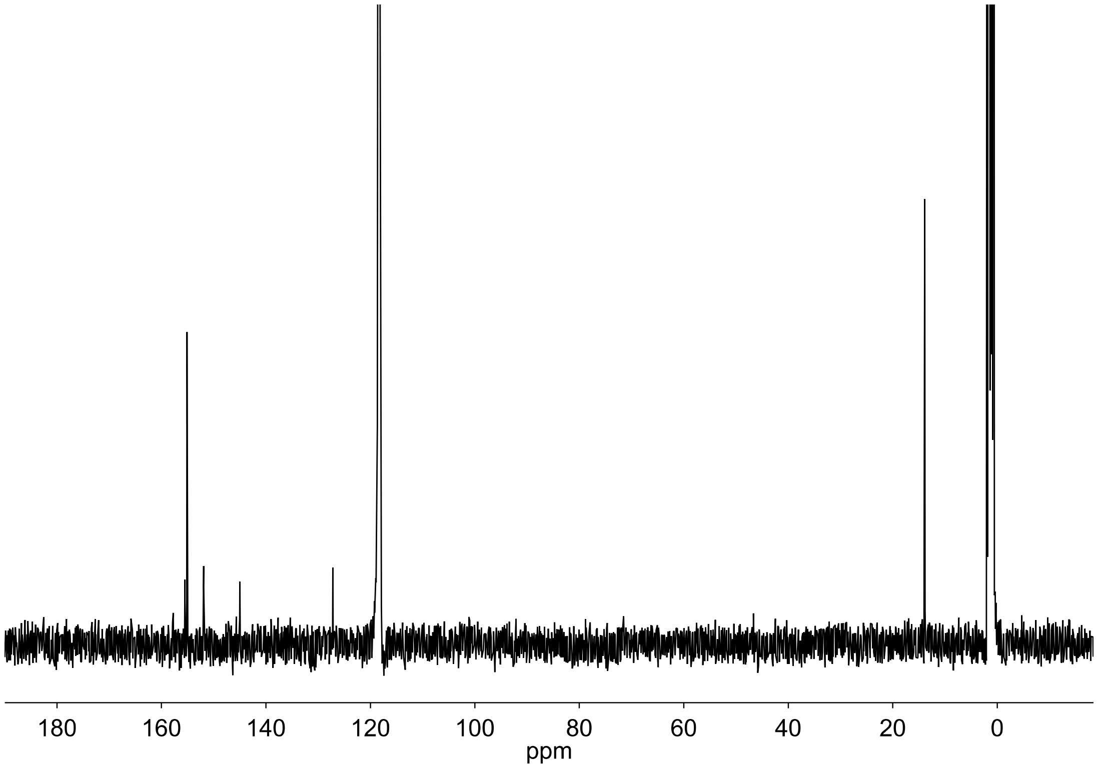

Figure S10. ^{13}C NMR spectrum of **3** recorded in CD_3CN.

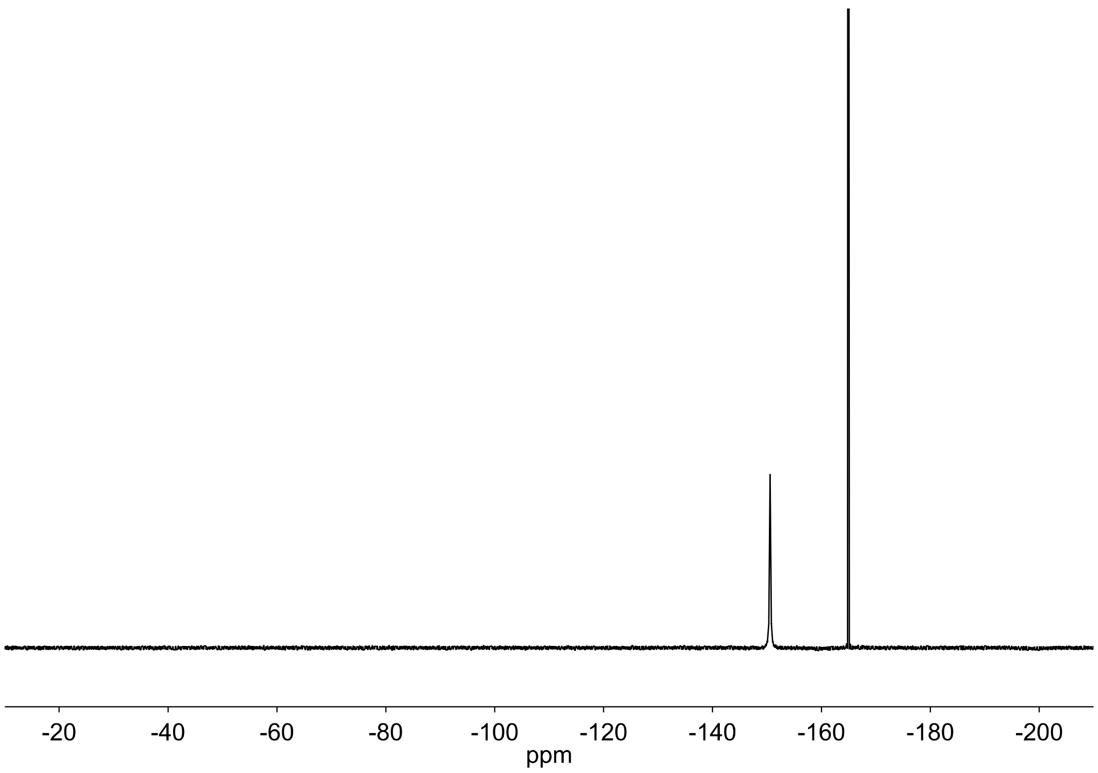

Figure S11. ^{19}F NMR spectrum of **3** recorded in CD_3CN.

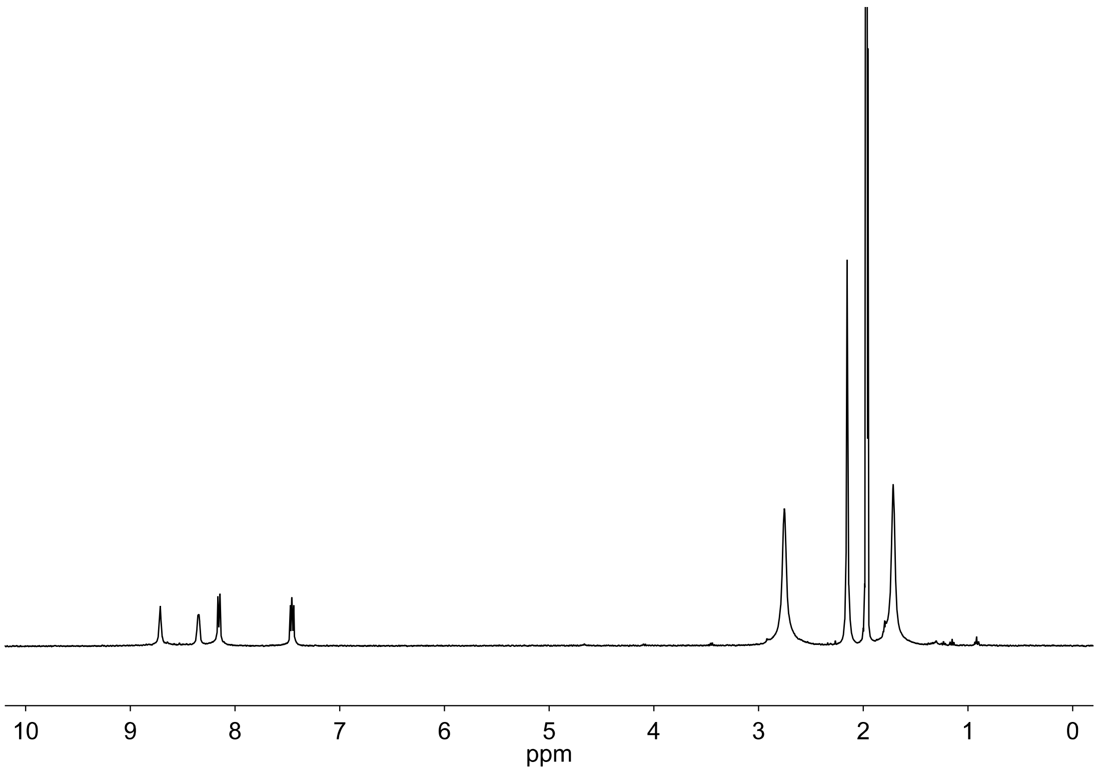

Figure S12. ^{1}H NMR spectrum of **4** recorded in CD_3CN.

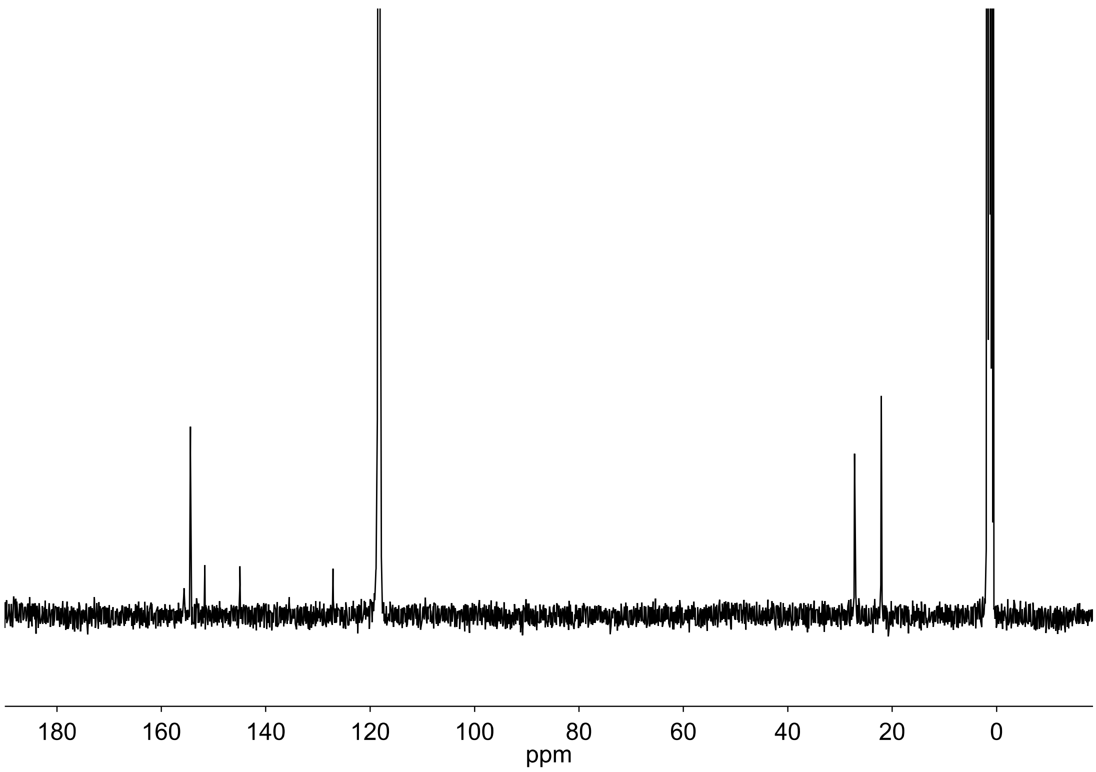

Figure S13. ^{13}C NMR spectrum of **4** recorded in CD_3CN.

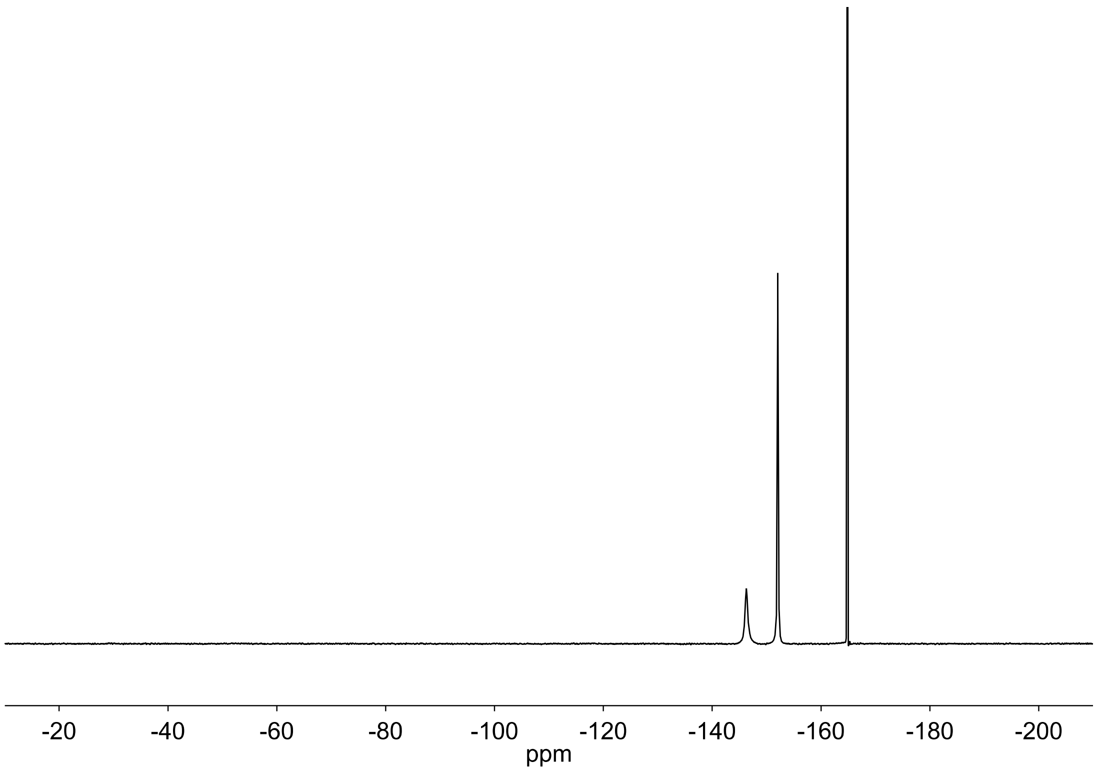

Figure S14. ^{19}F NMR spectrum of **4** recorded in CD_3CN.

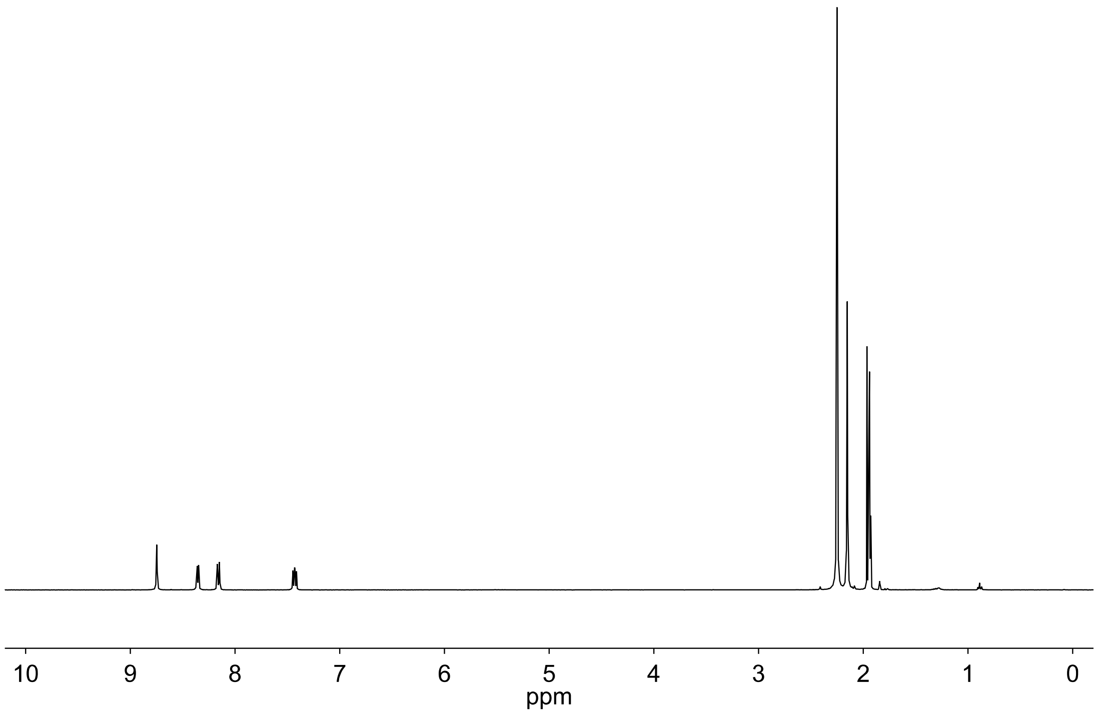

Figure S15. ^{1}H NMR spectrum of **5** recorded in CD_3CN.

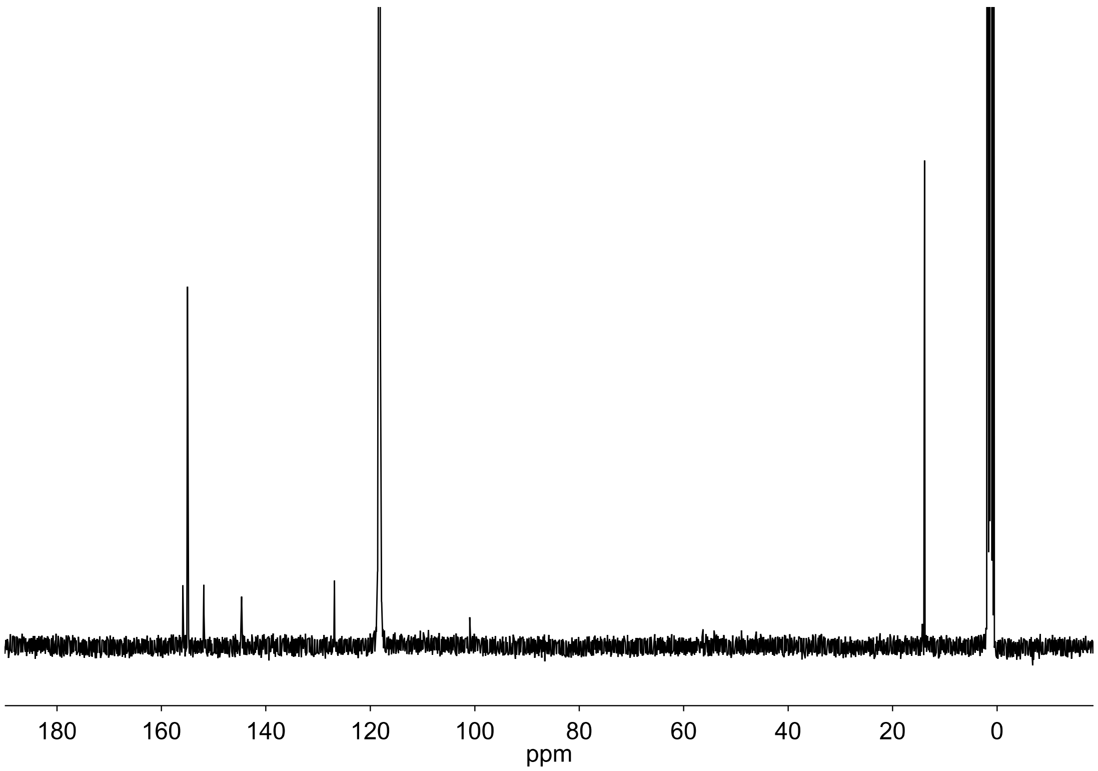

Figure S16. ^{13}C NMR spectrum of **5** recorded in CD_3CN.

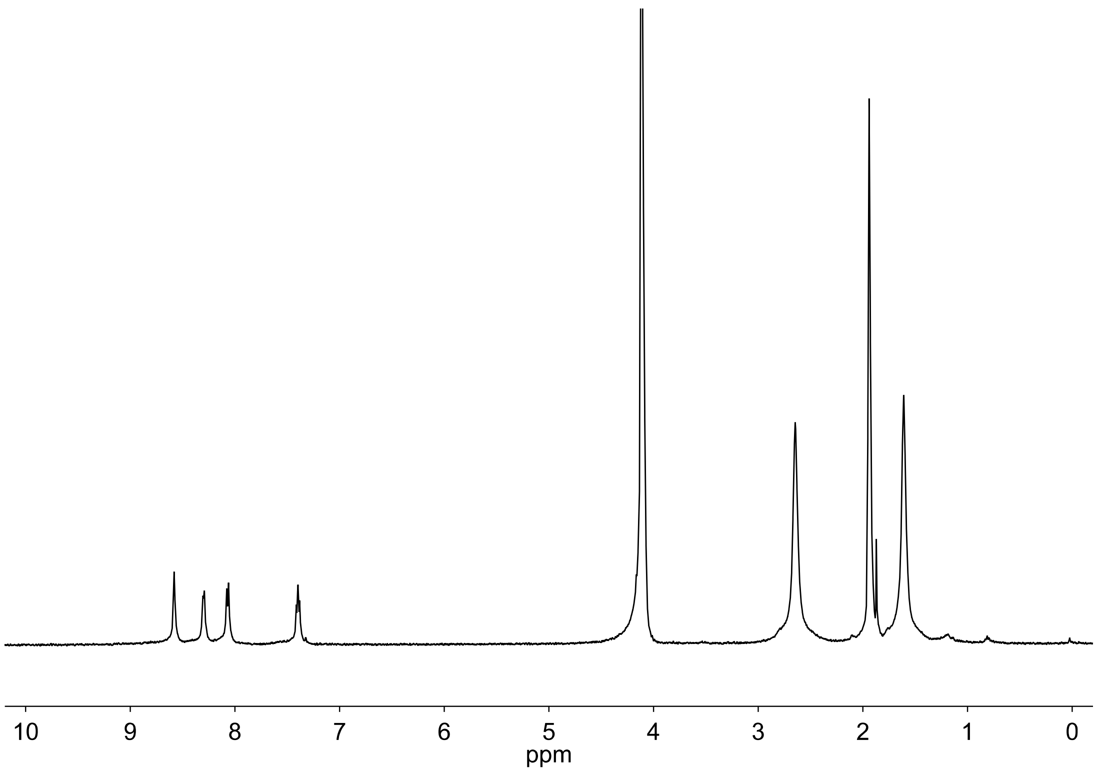

Figure S17. ^{1}H NMR spectrum of **6** recorded in a 5:1 mixture of CD_3CN:D_2O.

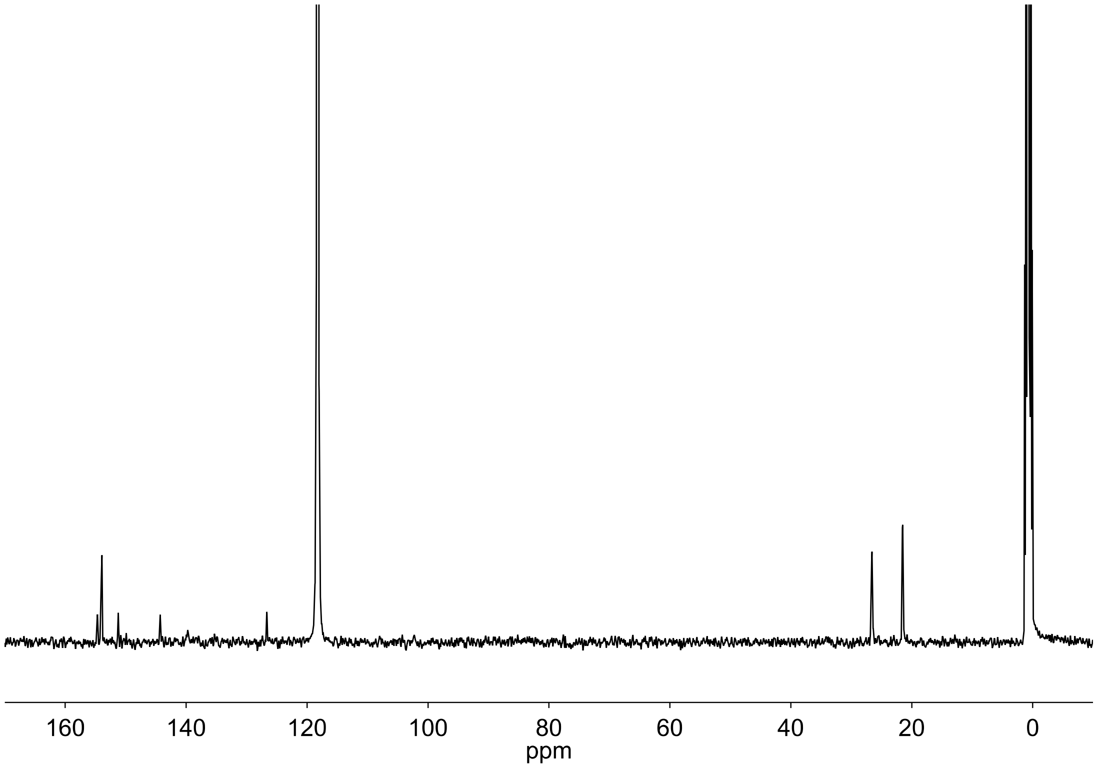

Figure S18. ^{13}C NMR spectrum of **6** recorded in a 5:1 mixture of CD_3CN:D_2O.

4.2 Monitoring the Formation of 3–6

The formation of **3–6** was monitored by ^1H NMR as follows. An NMR tube was charged with **1** or **2** (7.62 μmol), and CD_3CN (0.5 mL, for the preparation of **3** and **4**) or a 5:1 mixture of $CD_3CN:D_2O$ (0.5 mL, for the preparation of **5** and **6**) to give a pale orange suspension in all cases. Both **1** and **2** are sparingly soluble in these solvents, but solubility is sufficient to record a ^1H NMR spectrum. Subsequently, a solution of the appropriate Pd^{2+} salt (0.1 mL, 152.42 M, 2 equivalents) in either CD_3CN ($[Pd(CH_3CN)_4](BF_4)_2$ for the preparation of **3** and **4**) or 5:1 $CD_3CN:D_2O$ ($Pd(NO_3)_2(H_2O)_n$ for the preparation of **5** and **6**) was added to this suspension and the ^1H NMR spectrum was immediately recorded. The NMR tube was subsequently heated at 70 °C in an oil bath, removed at intervals and cooled to RT before the ^1H NMR spectrum was again recorded.

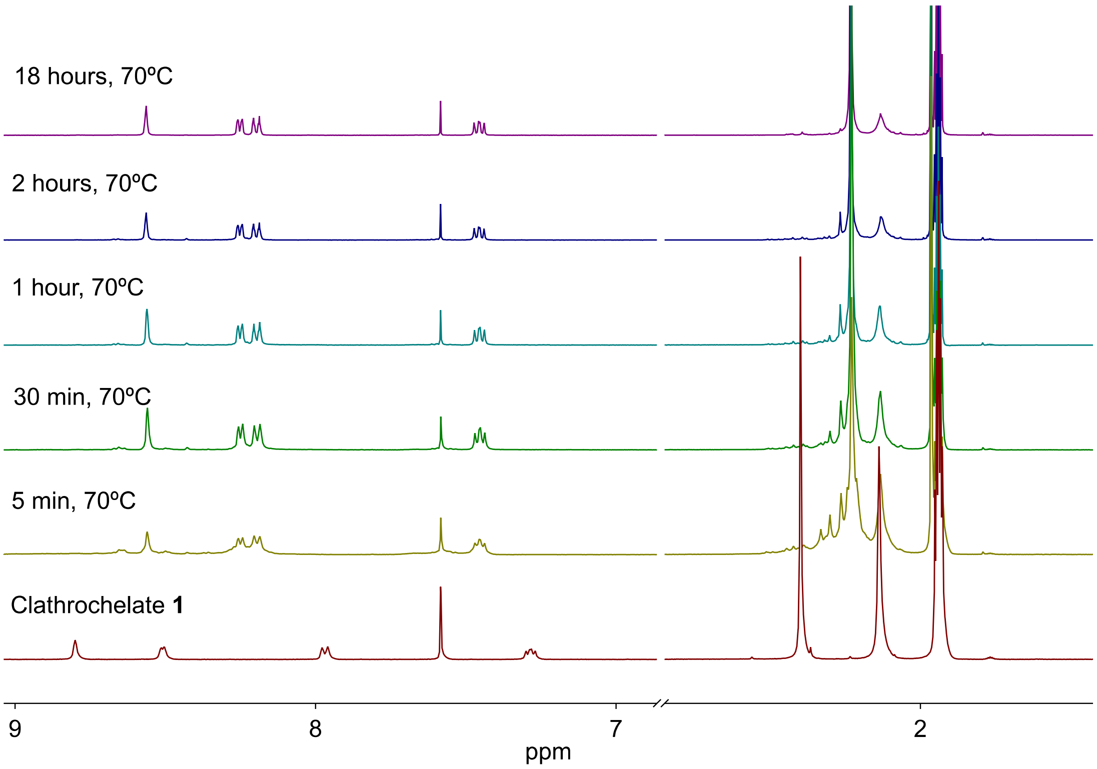

Figure S19. The formation of **3** monitored by ^1H NMR. Peaks at 1.94 ppm, 2.14 ppm and 7.58 ppm correspond to CHD_2CN, H_2O and $CHCl_3$ respectively.

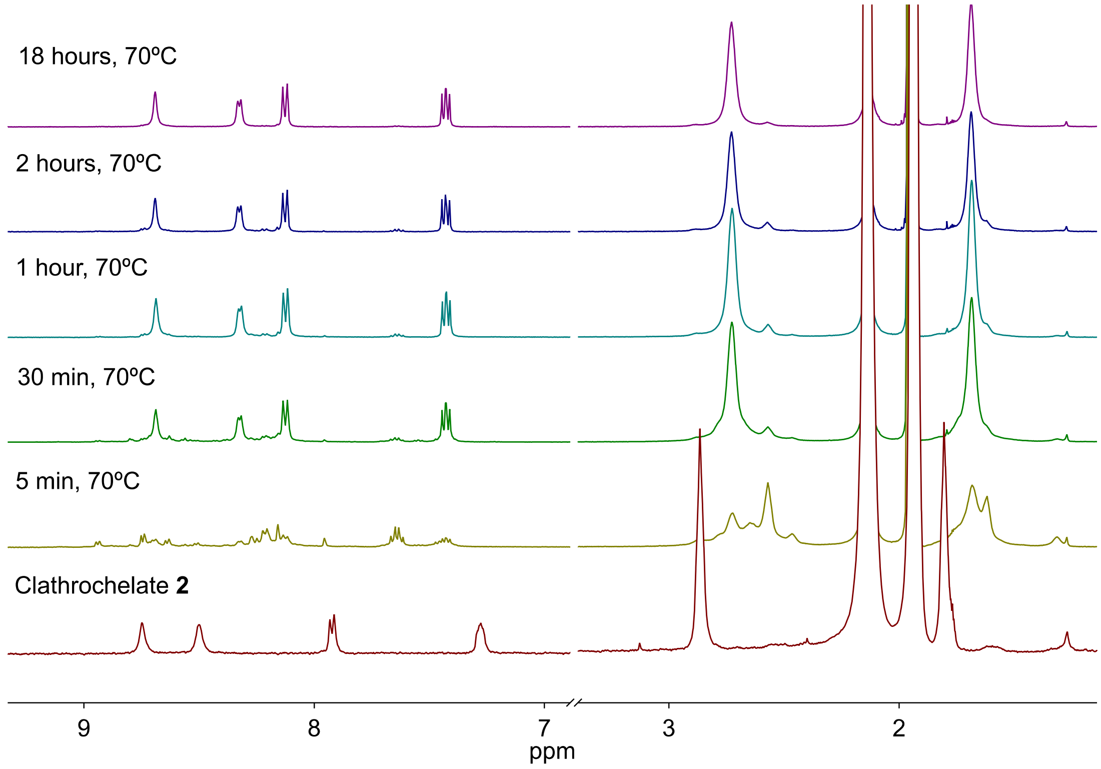

Figure S20. The formation of **4** monitored by ^{1}H NMR. Peaks at 1.94 ppm and 2.14 ppm correspond to CHD_2CN and H_2O respectively.

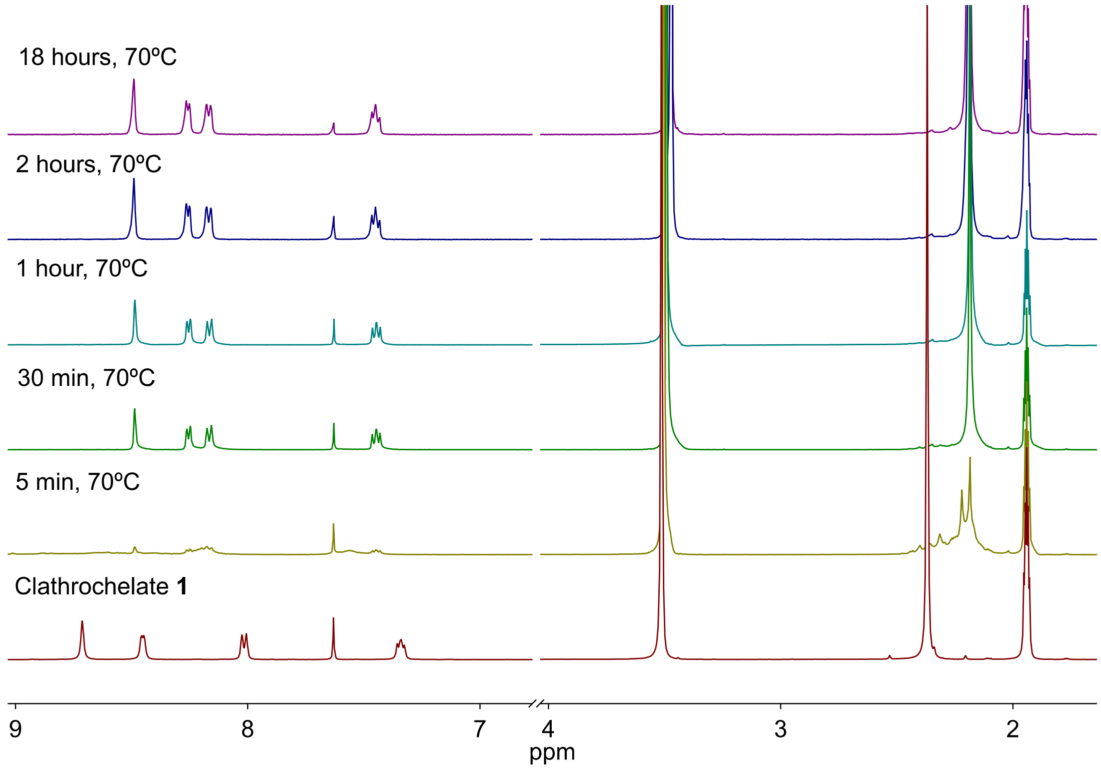

Figure S21. The formation of **5** monitored by ^{1}H NMR. Peaks at 1.94 ppm, 3.47–3.51 ppm and 7.63 ppm correspond to CHD_2CN, H_2O and $CHCl_3$ respectively.

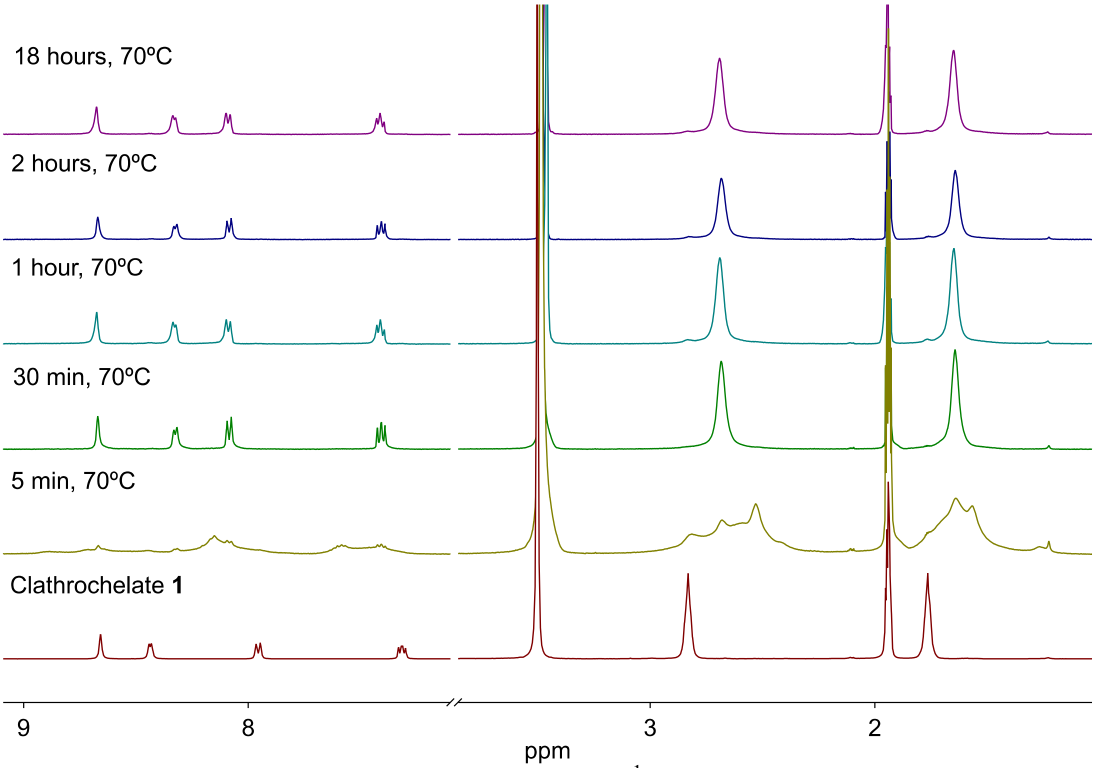

Figure S22. The formation of **6** monitored by ^{1}H NMR. Peaks at 1.94 ppm and 3.47–3.51 ppm correspond to CHD_2CN and H_2O respectively.

4.3 Low Temperature ^{1}H NMR Spectra of 3 and 4

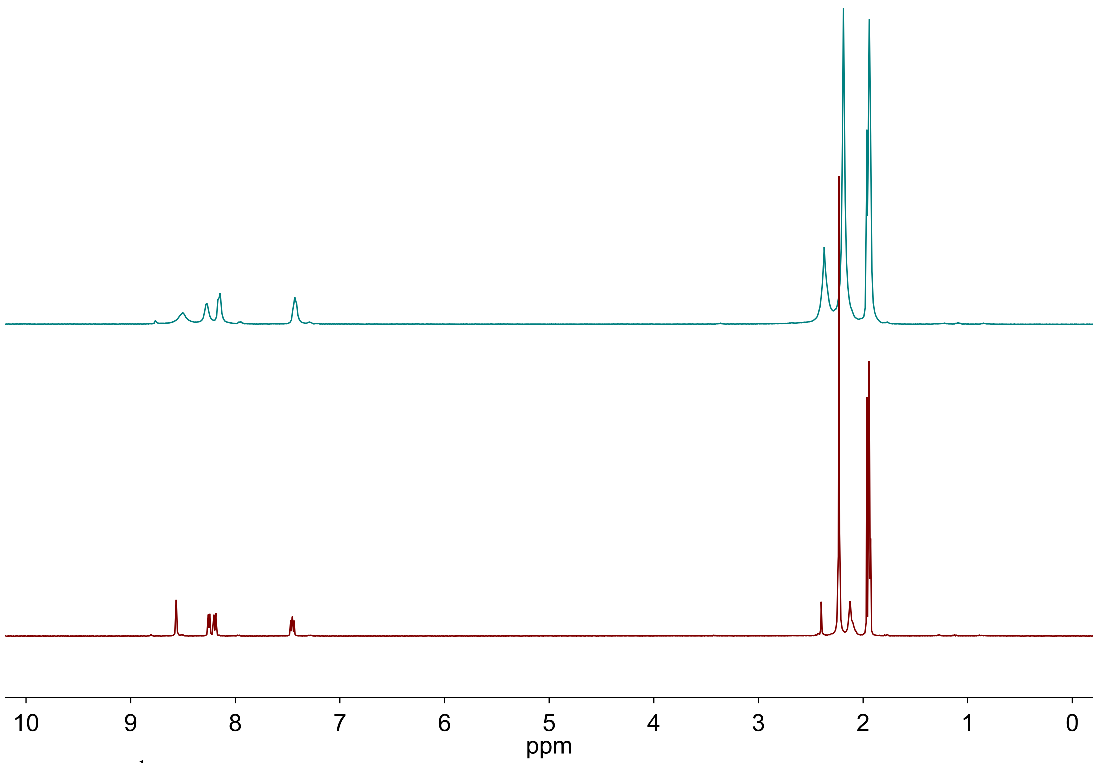

Figure S23. ^{1}H NMR spectrum (CD_3CN) of **3** recorded at 298K (bottom) and 233K (top).

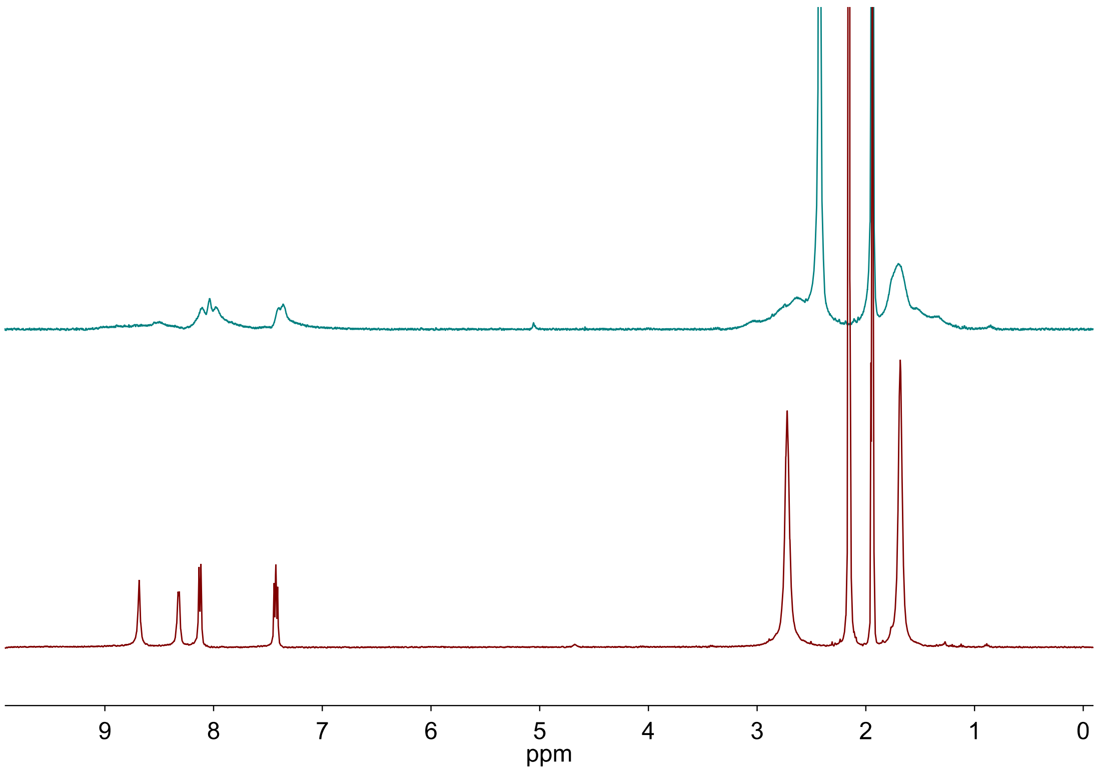

Figure S24. ^{1}H NMR spectrum (CD_3CN) of **4** recorded at 298K (bottom) and 233K (top).

4.4 Variable Temperature ^{19}F NMR Spectra of 3 and 4

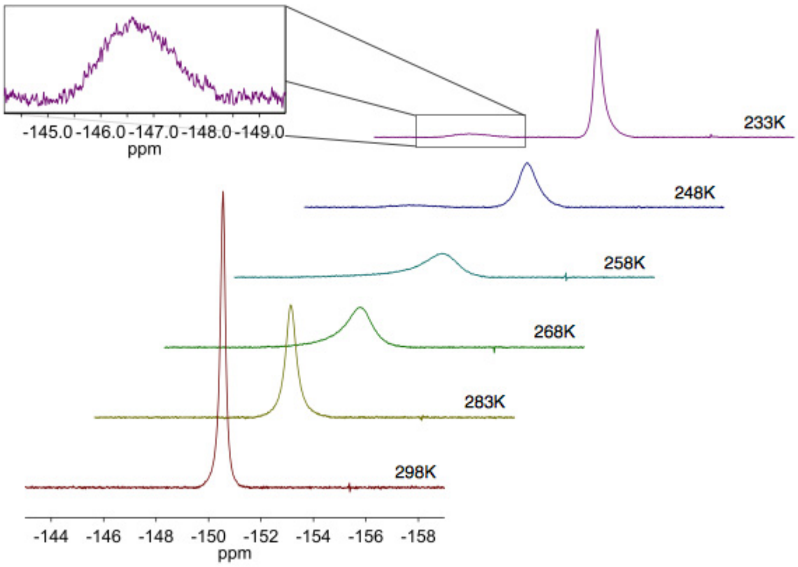

Figure S25. ^{19}F NMR spectrum of **3** recorded at selected temperatures in CD_3CN. The reference signal was used to reference to scale the intensities of the BF_4^- anion peaks.

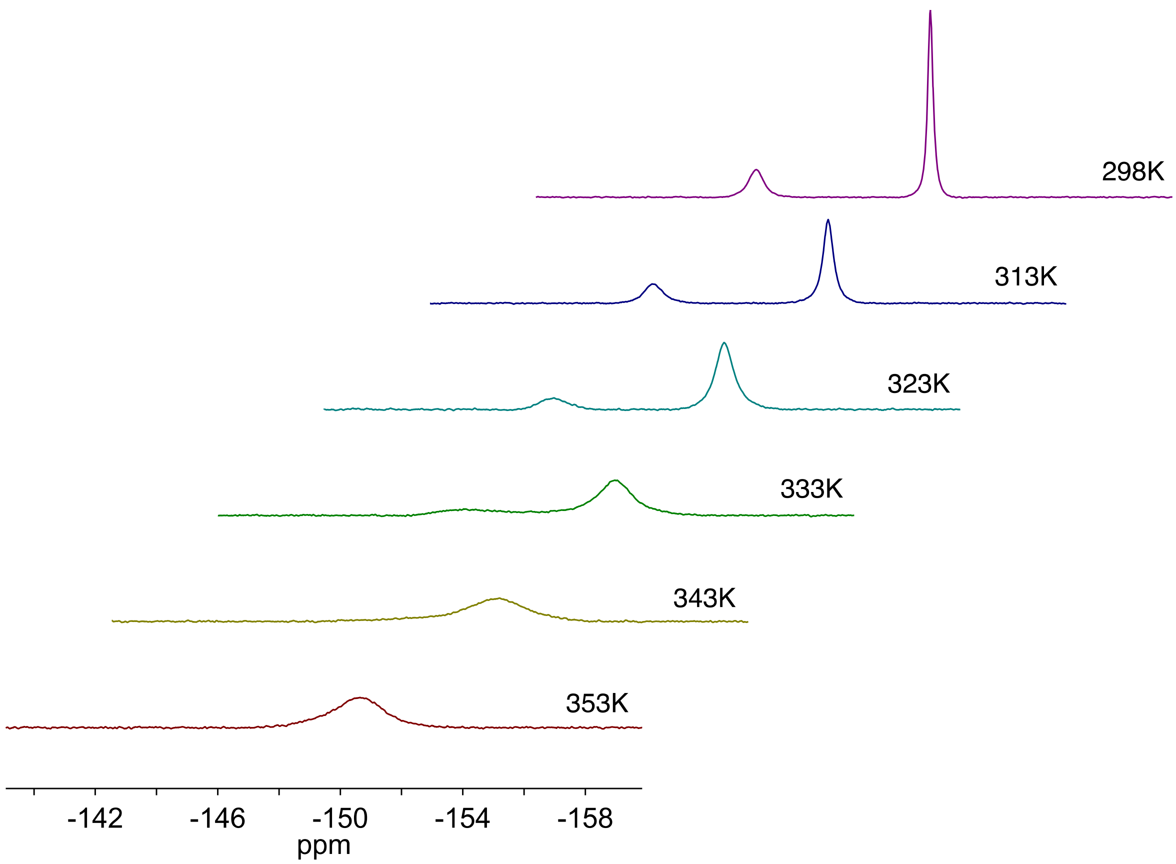

Figure S26. ^{19}F NMR spectrum of **4** recorded at selected temperatures in CD_3CN. The reference signal was used to scale the intensities of the BF_4^- anion peaks.

5. Single Crystal X-ray Analyses

5.1 General

Intensity data were collected at the Swiss Norwegian beamline BM01A at the ESRF in Grenoble (France) on the Pilatus@SNBL kappa goniometer from Huber Diffraktionstechnik GmbH equipped with a Pilatus2M pixel detector from Dectris Ltd, on an Oxford Diffraction KM-4 CCD diffractometer, a Bruker APEX II CCD system, or a marμx system. All data collections were performed at low temperature (100−140 K) using a Cryostream 700 Series from Oxford Cryosystems Ltd. Data integration was carried out using Crystalys Pro,[1] BYPASS,[2] EVALCCD,[3] automar[4] or XDS[5,6] in combination with autoPROC.[7] Multi-scan empirical absorption corrections were applied to the data using CrysAlis Pro or SADABS.[8] Data reduction was carried out using XPREP.[8] All structures were solved by direct methods or charge flipping using SIR,[9] SHELXT[10] or SUPERFLIP[11] and refined with SHELXL[10] using full-matrix least-squares routines on F^2 and ShelXle[12] as graphical user interface.

A series of carefully adapted macromolecular refinement techniques enabled us to successfully build and complete a molecular model. These methods already proved successful in previous cases of huge and complicated supramolecular structures with high solvent content.[13−15] All atoms were grouped into residues to enable addressing all atoms of repeating structural fragments with a single command. This grouping is required for the application of geometric restraint dictionaries generated with the Grade Program, which is part of BUSTER[16] and was accessed via the GRADE Web Server.[17] Grade restraint dictionaries were generated for clathrochelate ligands **1** and **2**, counterions and solvent molecules. In all cage structures a SHELX adapted version of local structural similarity restraints (LSSR) exploiting non-crystallographic symmetry (NCS)[18] were applied to additionally enhance similar geometries of each occurrence of clathrochelate ligands and counter ions. Rigid bond restraints (RIGU)[19] and isotropic restraints (ISOR) as well as similarity restraints (SIMU) were applied to the anisotropic displacement parameters of boron, carbon, nitrogen, oxygen and fluorine and chlorine atoms. Disordered moieties were placed into the asymmetric unit using the DSR program[20−21] in combination with a constrained rigid body refinement followed by a restrained refinement for which GRADE restraint dictionaries were used. Carbon-bound hydrogen atoms were included in idealized positions and refined using a riding model. The contribution of the electron density

associated with disordered counterions and solvent molecules, which could not be modelled with discrete atomic positions were handled using the SQUEEZE[22] routine in PLATON[23] and generated solvent masks (.fab files) were included in the SHELXL refinement via the ABIN instruction to leave the original structure factors untouched.

Crystallographic data have been deposited with the CCDC no. 1024735−1024740. Copies of the data can be obtained free of charge on application to the CCDC, 12 Union Road, Cambridge, CB2 1EZ, U.K. (fax, (internat.) +44-1223-336033; E-mail, deposit@ccdc.cam.ac.uk).

5.2 Ligands 1 and 2

Ligand 1: Data collection and refinement details

The asymmetric unit only contains half of the clathrochelate ligand **1**. It was modelled with rotational disorder using a free variable which resulted in an occupancy ratio of 0.659(3) : 0.341(3). Due to minor dynamic rotational disorder of chlorine atoms, which is reflected in their large ADPs, the carbon atom of the dichloromethane solvent had a significantly lower equivalent U value compared to chlorine atoms. The slightly too low goodness of fit most likely reflects a (tiny) overestimation of the diffraction data's sigma values.

Ligand 2: Data collection and refinement details

Due to the measurement on a single axis diffractometer, a completeness of only 92.9% for the triclinic space group *P*-1 could be achieved. Nevertheless, the overall structural quality is very good.

Table S1. Crystallographic data for ligand **1** and **2**.

Structure, CCDC no	Ligand 1, 1024735	Ligand 2, 1024736
Empirical formula	$C_{24}H_{28}B_2Cl_6FeN_8O_6$	$C_{31}H_{44}B_2Fe\ N_8O_9$
Mol. Weight / g mol^{-1}	814.71	750.21
Temperature / *K*	120(2)	140(2)
Wavelength / Å	1.54178	0.71073
Crystal system	Monoclinic	Triclinic
Space group	C2/c	P-1
a / Å	24.287(3)	10.435(7)
b / Å	8.1945(5)	12.522(6)
c / Å	20.3960(18)	14.568(8)
α / °	90	110.05(2)
β / °	120.269(13)	98.25(3)
γ / °	90	92.93(4)
Volume / Å^3	3505.8(7)	1759.4(18)
Z	4	2
Density / g cm^{-3}	1.544	1.416
Absorption Coeff. / mm^{-1}	8.090	0.493
Crystal size / mm^3	0.808 x 0.182 x 0.165	0.290 x 0.250 x 0.200
Θ range / °	4.215 to 73.272	2.278 to 25.345
Index ranges	$-29 \leq h \leq 29$, $-5 \leq k \leq 9$, $-16 \leq l \leq 25$	$-12 \leq h \leq 11$, $-15 \leq k \leq 15$, $-17 \leq l \leq 17$
Reflections collected	6161	11785
Independent reflections	3405 (R_{int} = 0.0434)	5977 (R_{int} = 0.0688)
Completeness	99.7 % (to Θ = 67.679°	92.9 % (to Θ =25.000°)
Absorption correction	Semi-empirical from equivalents	None
Max. & min. transmission	1.00000 and 0.02384	-
Data / restraints / param.	3405 / 88 / 257	5977 / 0 / 468
Goodness-of-fit on F^2	0.796	1.055
Final *R* indices [I > 2 s (I)]	*R1* = 0.0610, *wR2* = 0.1593	*R1* = 0.0741, *wR2* = 0.1923
R indices (all data)	*R1* = 0.0735, *wR2* = 0.1741	*R1* = 0.1129, *wR2* = 0.2164
Extinction coefficient	0.00039(8)	-
Larg. diff. peak/hole / eÅ^{-3}	0.703 and -0.615	0.576 and -0.537

5.3 Assemblies 3, 5, 6 and 7

5.3.1 Cage 3: Data collection and refinement details

The X-ray diffraction measurements were carried out on the Swiss Norwegian beamline (BM01A) at the ESRF in Grenoble, France. The incident synchrotron beam from a bending magnet source was focused onto the sample using a combination of curved mirrors and a sagitally bent Si(111) monochromator crystal. The focused beam had a wavelength of 0.7150Å and a FWHM at the sample position of about 0.5mm.

The crystals employed in this study rapidly suffered solvent loss. The samples were selected and handled under cryogenic conditions and protected by liquid nitrogen to prevent loss of solvent and reaction with the atmosphere. A lustrous red crystal with a pyramidal shape and a diameter of about 0.2mm was chosen for data collection of cage 3 (Figure S27).

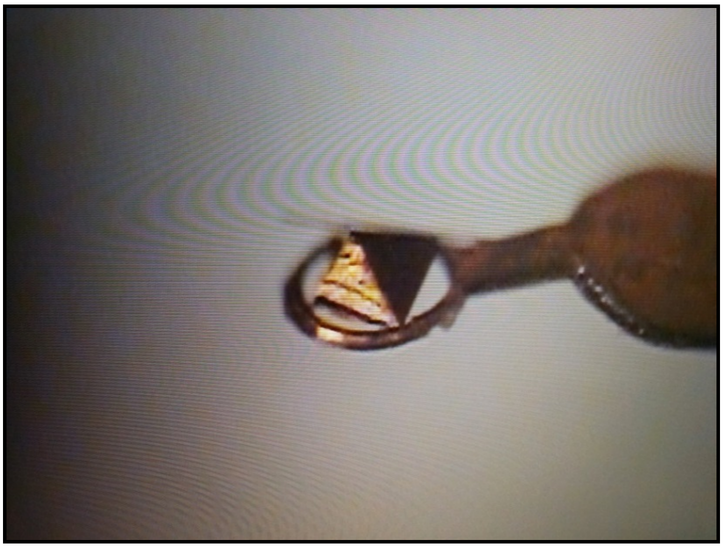

Figure S27. The selected crystal mounted on a 0.2mm diameter cryoloop.

A total of 3000 images in various kappa orientations were collected in omega slices of 0.25° width and an exposure time of 1s per image. The sample-to-detector distance was 194.3mm and a sub-atomic resolution of 0.84 Å was reached.

Data analysis was carried out using the CrysalisPro software package (version 171.35.21) from Agilent Ltd. A structure solution was found using charge flipping as implemented in the Superflip software[11] in the space group F432. Two crystallographically independent cages are present in this model. The first one (Cage 3A) has 4-fold crystallographic

symmetry. The second one (Cage 3B) has 23 crystallographic symmetry and is representing an ideal octahedron. The structure was refined as an inversion twin and the flack parameter value was 0.50(2) but attempts to refine the structure in the centrosymmetric space-group Fm-3m were not successful. The site occupancy factor of one diethylether solvent molecule and one BF_4^- anion being close to each other were fixed to 0.5 and atomic displacement parameters of both species were refined only isotropically. There is a significant amount of thermal motion in the extremities of the molecule. Even with the applied restraints for displacement parameters, described in the general section, equivalent U values of neighboring atoms were not ideal in some of the cases. Mainly atoms belonging to or near to disordered areas were affected. Disordered solvent/anion areas occupy 45% of the unit cell volume, which accounts for the low calculated crystallographic density and also reflects the solvent loss. The area of diffuse electron density could not be successfully modeled with discrete atomic positions and the SQUEEZE[22] function of PLATON[23] was employed. Nevertheless, high residuals close to palladium atoms remain. Due to the large unit cell volume and amount of collected data at the synchrotron summing up to 100 MB of unmerged diffraction data, the "shelx hkl file record" needed to be removed from the CIF file before running the server-based CHECKCIF to avoid a timeout during the upload.

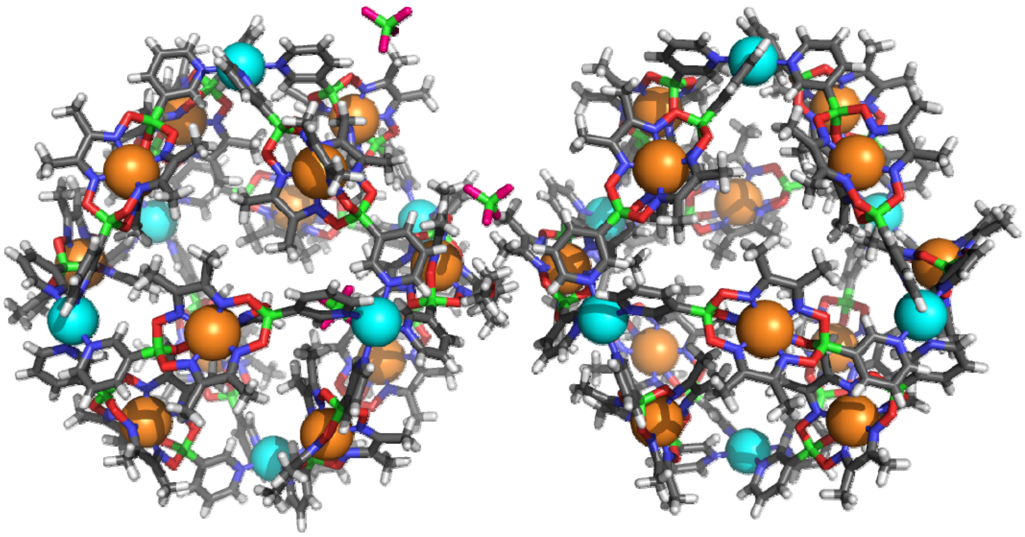

Figure S28. Atomic representation of the asymmetric unit of cage **3** showing both cages, and ordered Et_2O solvent and BF_4^- counter ions.

Cage 5: Data collection and refinement details

The X-ray diffraction measurements were carried out on the Swiss Norwegian beamline (BM01A) at the ESRF in Grenoble, France and a sub-atomic resolution of 0.74 Å was reached. Raw data were processed using XDS[5,6] in combination with autoPROC.[7] Disorder

of one clathrochelate ligand (residue 6) and three toluene solvent molecules (residue 21, 25 and 26) were modelled using free variables which resulted in an occupancy ratio of 0.652(3):0.348(3) different parts of residue 6, 0.546(10):0.454(10) for residue 21, 0.759(8):0.241(8) for residue 25 and 0.652(3):0.348(3) for residue 26. Due to the disorder, the displacement parameters of atoms in residue 25 were only refined isotropically. There is a significant amount of thermal motion in the extremities of the molecule and modelled solvents. Even with the applied restraints for displacement parameters, described in the general section, equivalent U values of neighboring atoms were not ideal in some of the cases. Mainly atoms belonging to or near to disordered areas were affected. Disordered solvent/anion areas occupy 13% of the unit cell volume. The area of diffuse electron density could not be successfully modeled with discrete atomic positions and the SQUEEZE[22] function of PLATON[23] was employed.

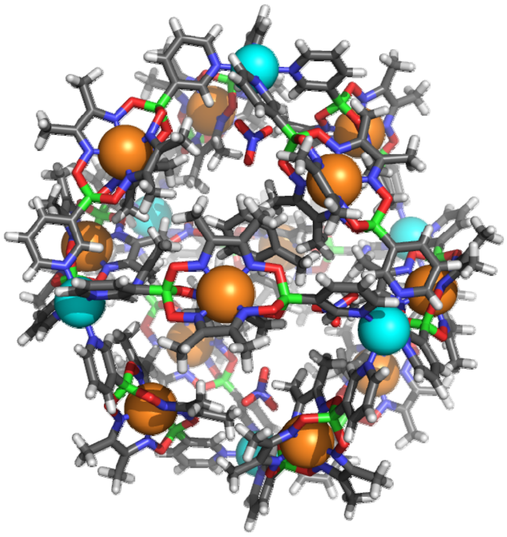

Figure S29. Atomic representation of the asymmetric unit of cage **5** showing ordered encapsulated toluene and acetonitrile solvent molecules as well nitrate counterions.

Cage 6: Data collection and refinement details

The X-ray diffraction measurements were carried out in-house and few reflections greater than 1.2 Å resolution were observed. There are two short contacts. It is very likely that there are several (dynamically disordered) conformations of the affected moieties, which cannot be properly modelled at this (1.2 Å) resolution. There also is a significant amount of thermal motion in the extremities of the molecule and modelled solvents. Even with the applied restraints for displacement parameters, described in the general section, equivalent U values of neighboring atoms were not ideal in some of the cases. Mainly atoms

belonging to or near to disordered areas were affected. Disordered solvent/anion areas occupy 56% of the unit cell volume. The area of diffuse electron density could not be successfully modeled with discrete atomic positions and the SQUEEZE[22] function of PLATON[23] was employed.

Although indicated in CHECKCIF alerts, we do not consider large Hirshfeld differences between metal ions and light atoms (C,N,O) problematic. The Hirshfeld test validates, if the contribution of the (anisotropic) displacement parameters (otherwise called B factors) is similar in bond direction of two bonded atoms, but can only be sensibly applied for atoms of similar size (weight), which is why restraints like RIGU for metal ions and their coordinated atoms of the ligands were intentionally avoided. This however results in this large list of insensible alerts, which are very likely to also occur for small molecule structures with the same structural motif, but due to a smaller number of total atoms in the structure a shorter list would appear to be less dramatic.

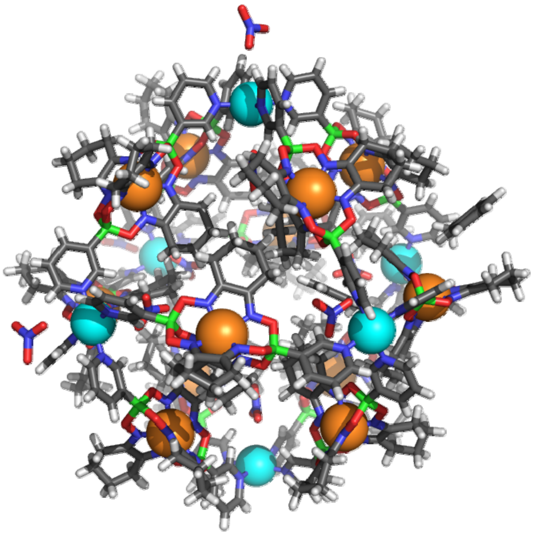

Figure S30. Atomic representation of the asymmetric unit of cage **6** showing the octahedral cage, encapsulated toluene solvent molecule and nitrate counter ions.

Cage 7: Data collection and refinement details

The X-ray diffraction measurements were carried out in-house and few reflections greater than 1.4 Å resolution were observed. The structure was solved using charge flipping as implemented in the Superflip software.[11] The available data did not allow for exhaustive modelling of finer structural details like (restrained) anisotropic displacement parameters of the 1392 independent non-hydrogen, location and placement of hydrogens at any of the 21 modelled water molecules or describing parts of disorder with several atomic positions.

There are a few short contacts. Apart from observed electron density supporting the molecular model (see Figure S32) there is however a large flexibility in these regions. It is very likely that there are several (dynamically disordered) conformations of the affected moieties, which cannot be properly modelled at this (1.4 Å) resolution.
There is a significant amount of thermal motion in the extremities of the molecule and modelled solvents. Even with the applied restraints for displacement parameters (SIMU), equivalent U values of neighboring atoms were not ideal in some of the cases. Mainly atoms belonging to or near to disordered areas were affected. Disordered solvent/anion areas occupy 27% of the unit cell volume. The area of diffuse electron density could not be successfully modeled with discrete atomic positions and the SQUEEZE[22] function of PLATON[23] was employed.
This structure is similar to a macromolecular type of structure (e.g. proteins) in terms of weak scattering power and resulting data quality (e.g high R_{int} and $wR2$) as well as unit cell volume and number of independent atoms, and the adapted macromolecular refinement techniques described in the general part were key to establish the connectivity of all modeled structural components with reasonably good precision.
Although CHECKCIF criteria are valid and necessary to assess the quality of small molecule structures, some of the criteria (e.g. requirement of atomic resolution) are unsuited for structures of the macromolecular domain and should be replaced with more sensible ones for supramolecular structures with these characteristics. In this context, it should also be mentioned that the current version of CHECKCIF installed on the IUCR web server (checkcif.iucr.org/) cannot cope with structures of this size. We have therefore only been able to run an updated local version of CHECKCIF routines on this structure. This updated version of CHECKCIF has kindly been provided by Prof. Dr. Anthony L. Spek.

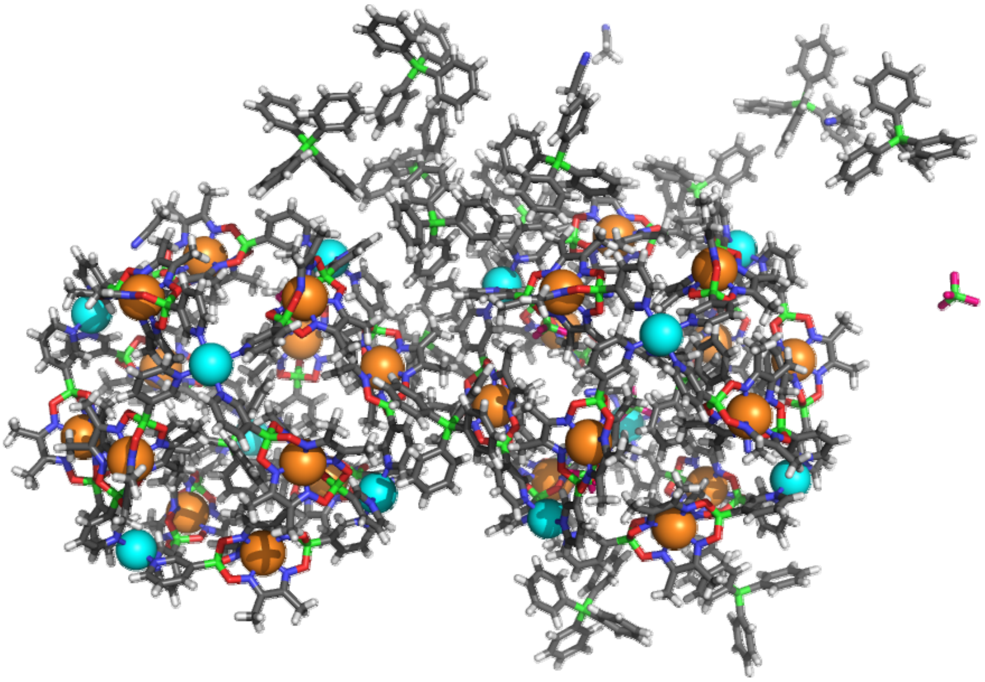

Figure S31. Atomic representation of the asymmetric unit of **7** showing both cages, acetonitrile and water solvent molecules as well as tetrafluoroborate and tetraphenylborate counter ions.

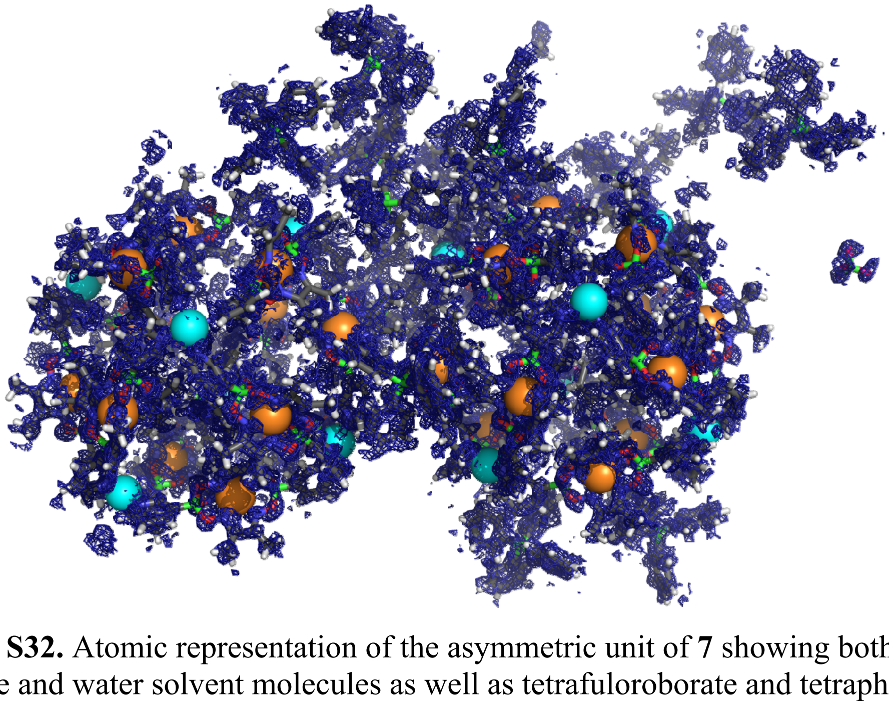

Figure S32. Atomic representation of the asymmetric unit of **7** showing both cages, acetonitrile and water solvent molecules as well as tetrafuloroborate and tetraphenylborate counter ions. In addition to the previous figure the observed electron density (F_O) is shown as a blue mesh at 1.0 σ level.

Table S2. Crystallographic data for cages **3**, **5, 6** and **7**

**Structure, CCDC no**	**3, 1024737**	**5, 1024738**	**6, 1024740**	**7, 1024739**
Empirical formula	$C_{270}H_{327}B_{29.5}F_{22}Fe_{12}N_{96}O_{73.5}Pd_6$	$C_{193}H_{230}B_{12}Fe_6N_{59}O_{53}Pd_3$	$C_{350}H_{400}B_{24}Fe_{12}N_{105}O_{99}Pd_6$	$C_{907}H_{953.5}B_{67.5}F_{18}Fe_{24}N_{201.5}O_{167}Pd_{12}$
Mol. Weight / g mol^{-1}	8138.75	5008.37	9229.77	21038.03
Temperature / *K*	100(2)	100(2)	140(2)	100(2)
Wavelength / Å	0.7150 (synchrotron)	0.7150 (synchrotron)	1.54178	0.71073
Crystal system	Cubic	Triclinic	Triclinic	Triclinic
Space group	F432	P-1	P-1	P-1
a / Å	77.1812(1)	24.070(5)	26.3697(6)	37.76(2)
b / Å	77.1812(1)	25.090(5)	37.7311(9)	37.914(15)
c / Å	77.1812(1)	26.260(5)	41.4010(16)	49.64(3)
α / °	90	111.25(3)	113.826(3)	109.87(3)
β / °	90	101.84(3)	96.680(2)	111.57(4)
γ / °	90	110.60(3)	90.021(2)	90.72(4)
Volume / Å^3	459763.6(18)	12790(6)	37375(2)	61382(56)
Z	32	2	2	2
Density / g cm^{-3}	0.941	1.300	0.820	1.138
Absorption Coeff. / mm^{-1}	0.536	0.615	3.362	0.514
Crystal size / mm^3	0.240 x 0.130 x 0.080	0.200 x 0.100 x 0.050	0.150 x 0.100 x 0.060	0.311 x 0.234 x 0.086
Θ range / °	1.570 to 25.076	0.905 to 28.755	3.259 to 42.094	1.075 to 17.200
Index ranges	$-90 \leq h \leq 83$<br>$-90 \leq k \leq 85$<br>$-91 \leq l \leq 85$	$-32 \leq h \leq 32$<br>$-30 \leq k \leq 33$<br>$-35 \leq l \leq 35$	$-22 \leq h \leq 21$<br>$-32 \leq k \leq 32$<br>$-35 \leq l \leq 35$	$-31 \leq h \leq 31$<br>$-31 \leq k \leq 30$<br>$-41 \leq l \leq 41$
Reflections collected	795832	877170	117057	222504
Independent reflections	32544 [R(int) = 0.0379]	60736 [R(int) = 0.0680]	51393 [R(int) = 0.0836]	72139 [R(int) = 0.1885]
Completeness	98.0 % (to Θ = 25.077°)	98.3 % (to Θ =25.077°)	99.9 % (to Θ = 42.000°)	99.8 % (to Θ = 17.000°)
Absorption correction	Semi-empirical from equivalents	none	Semi-empirical from equivalents	Semi-empirical from equivalents
Max. & min. transmission	1.00000 and 0.93086	-	1.00000 and 0.55382	0.6993 and 0.5760
Data / restraints / param.	32544 / 4154 / 1526	60736 / 9072 / 3304	51393 / 15269 / 5365	72139 / 42737 / 5610
Goodness-of-fit on F^2	1.300	1.021	1.299	1.394
Final *R* indices [I > 2 s (I)]	*R1* = 0.0810, *wR2* = 0.2584	*R1* = 0.0557, *wR2* = 0.1600	*R1* = 0.1105, *wR2* = 0.3243	*R1* = 0.1670, *wR2* = 0.3795
R indices (all data)	*R1* = 0.0829, *wR2* = 0.2646	*R1* = 0.0593, *wR2* = 0.1690	*R1* = 0.1500, *wR2* = 0.3519	*R1* = 0.2907, *wR2* = 0.4645
Extinction coefficient	-	-	-	-
Larg. diff. peak/hole / eÅ^{-3}	3.178 and -0.583	2.523 and -1.249	1.163 and -0.722	3.608 and -1.283
Flack x (Parsons)	0.50(2)	-	-	-

5.3 Cavity Volume Calculations

Crystallographically determined octahedral cages of structures **3**, **5**, **6** and **7** were symmetry expanded (if required) and guests molecules as well as counter ions were removed. Resulting inner cavities were calculated with VOIDOO,[24] using a primary grid and plot grid spacing of 0.2 Å and ten cycles of volume refinement. To prevent the probe from "escaping" the inner sphere through the large pores, the default water size probe radius of 1.4 Å was increased to 1.7 Å for all structures. This procedure result in smaller calculated volumes compared to using the default probe size, but allows for a systematic comparison of resulting cavity volumes.

For both independent cages of structure **7** the cavities are visualized as a 50% transparent blue iso surface inside the crystal. Molecular visualization was done using PyMol.[25]

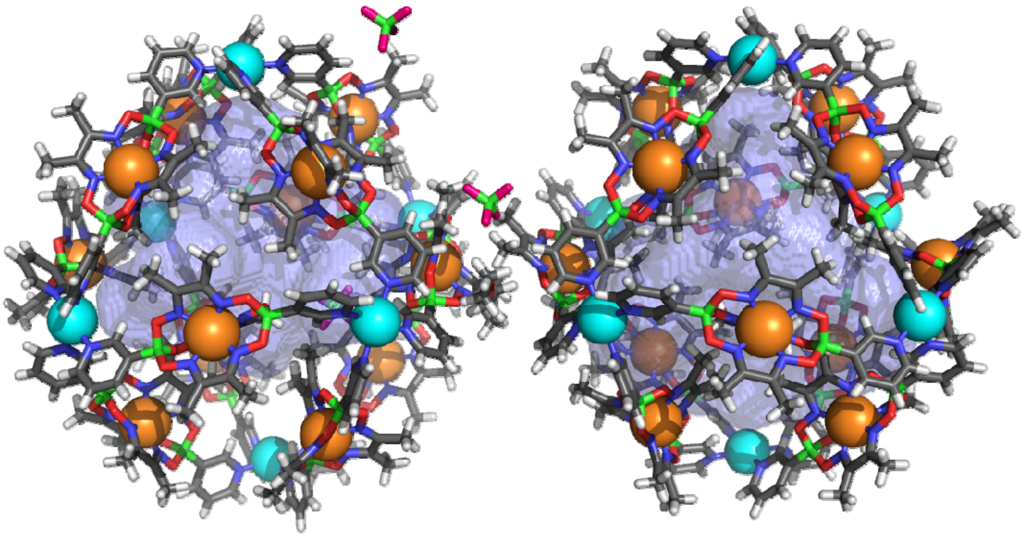

Figure S33. Cavity visualization for both octahedral cages in the cage **3** structure. Cavities are shown as a 50% transparent blue iso surface.

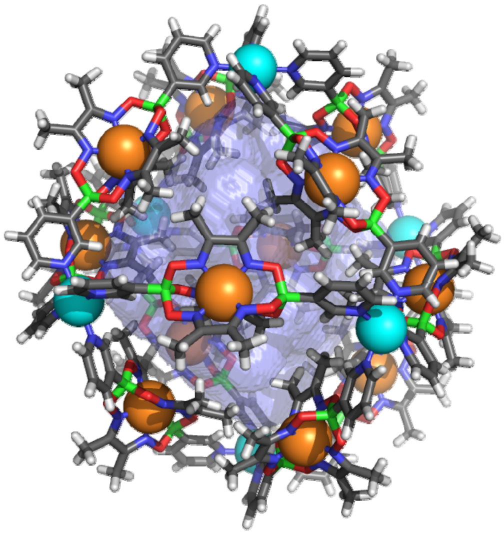

Figure S34 Cavity visualization for assembly **5**. Cavity shown as a 50% transparent blue iso surface. Encapsulated solvent molecules as well as counterions have been omitted.

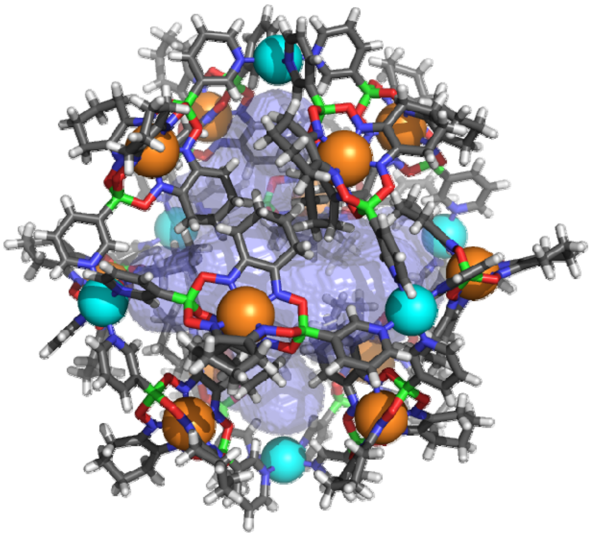

Figure S35 Cavity visualization for both assembly **6**. Cavities are shown as a 50% transparent blue iso surface. Solvent molecules as well as counterions have been omitted.

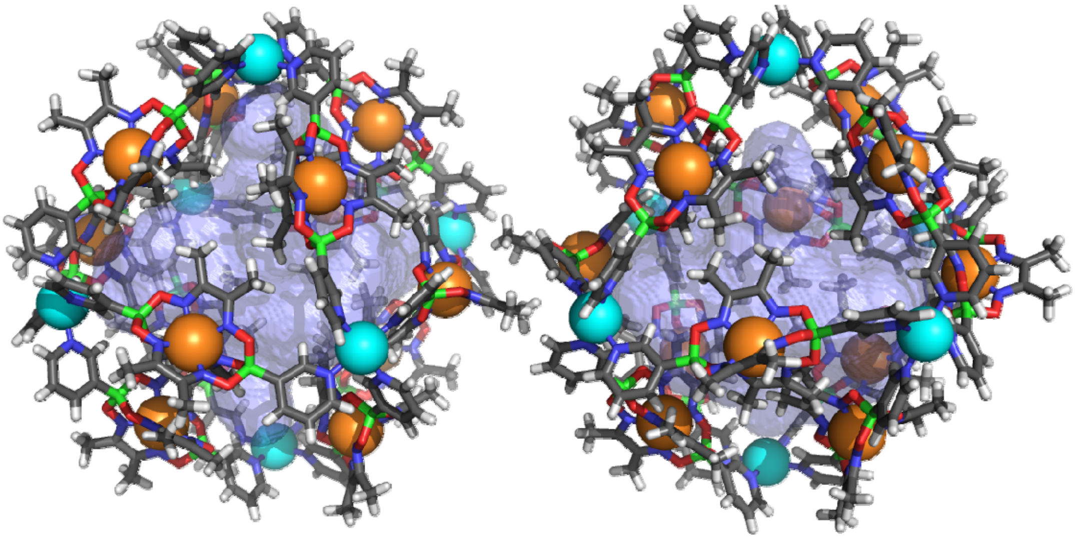

Figure S36 Cavity visualization for both octahedral cages in **7**. Cavities are shown as a 50% transparent blue iso surface. Encapsulated acetonitrile solvent molecules as well as anions have been omitted.

6. Encapsulation of the BPh_4^- Anion

6.1 Experimental Methodology

Experiments to elucidate the interaction of cage **3** with the BPh_4^- anion were undertaken as follows. An NMR tube was charged with **3** (2.7 mg, 0.313 μmol) and CD_3CN (0.5 mL). Subsequently, 10 μL aliquots of a solution of $NaBPh_4$ in CD_3CN (10.7 gL^{-1}, 31.3 mM) were added sequentially and the sample left at RT for 12 h after each addition in order to ensure equilibrium had been attained. DOSY and NOESY NMR spectra were recorded after this 12 h period. The methodology was identical for initial binding experiments undertaken in 2:1 CD_3CN:H_2O and 1:1 CD_3CN:$CDCl_3$. However, in order to acquire spectra of sufficient resolution to integrate peaks corresponding to free BPh_4^- in 2:1 CD_3CN:H_2O, the initial amount of **3** was reduced to 1.4 mg.

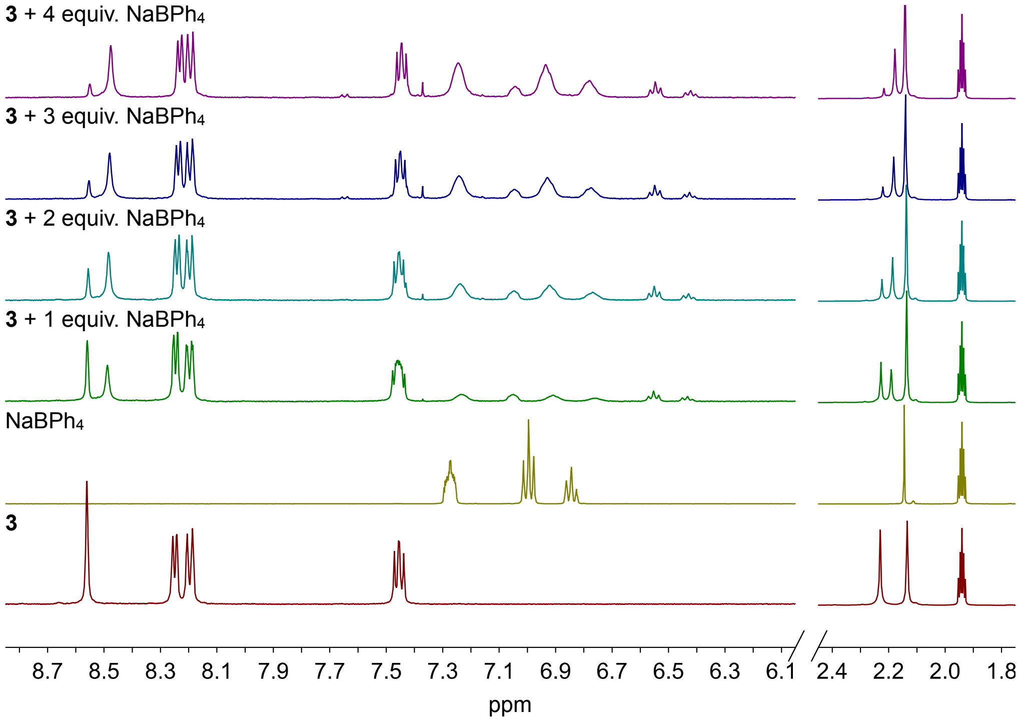

Figure S37. Changes in the ^{1}H NMR spectrum of **3** (bottom, red) in CD_3CN upon addition of $NaBPh_4$ (second from bottom, yellow). For clarity, the aromatic region has been scaled to a much higher intensity than the aliphatic region.

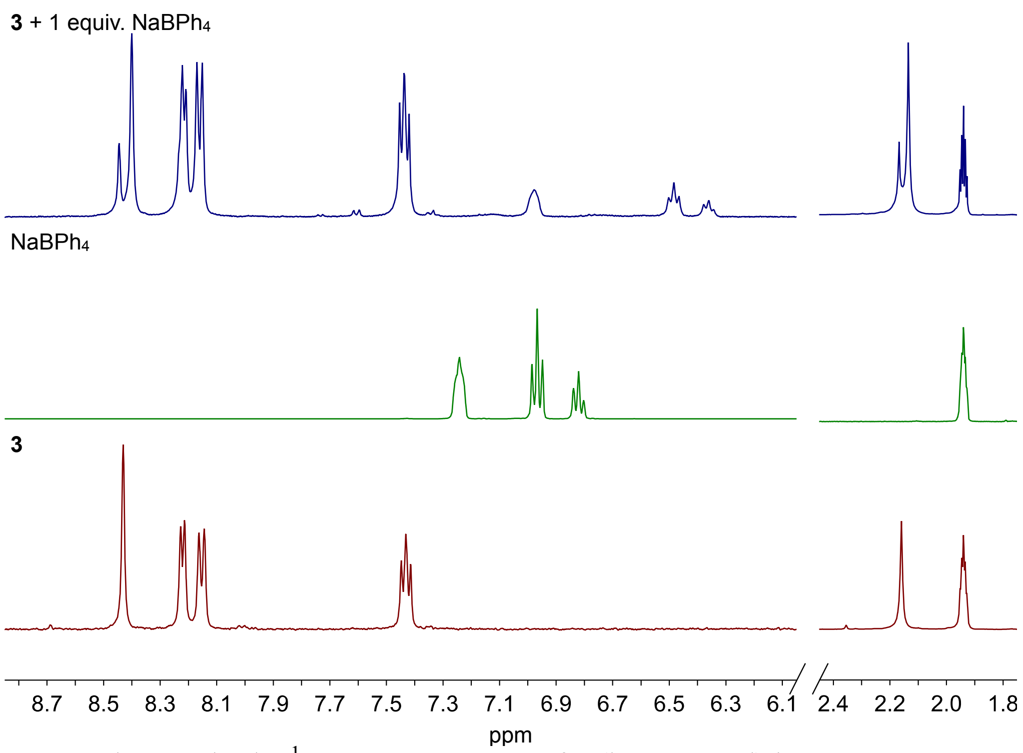

Figure S38. Changes in the ^{1}H NMR spectrum of **3** (bottom, red) in 2:1 CD_3CN:D_2O upon addition of $NaBPh_4$ (second from bottom, yellow). For clarity, the aromatic region has been scaled to a much higher intensity than the aliphatic region.

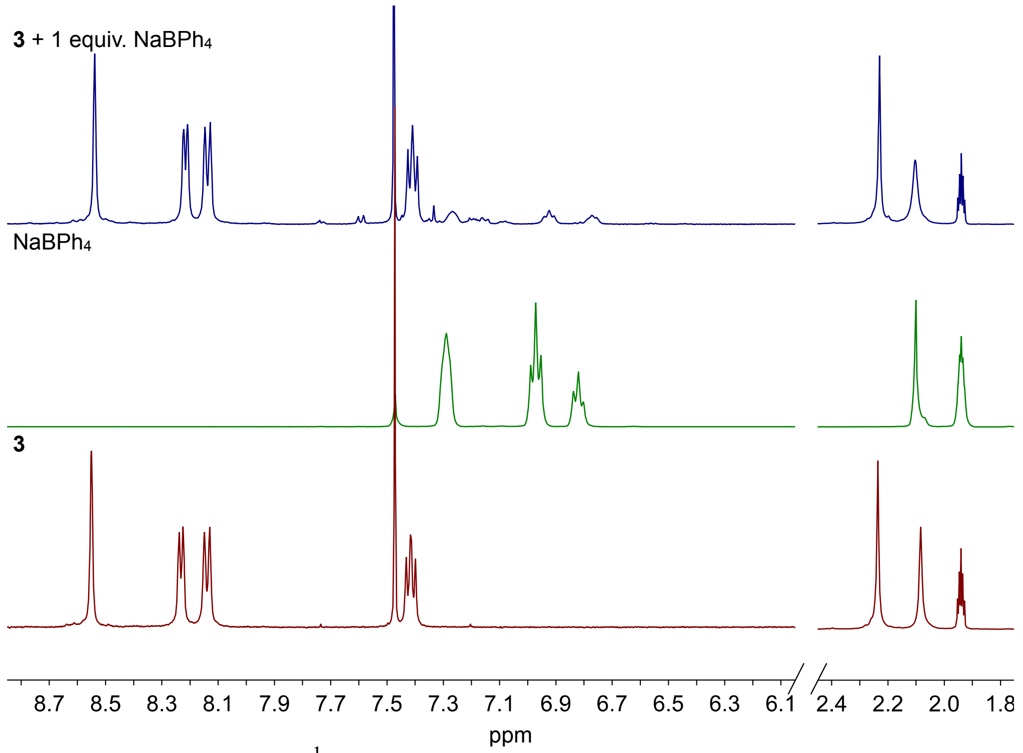

Figure S39. Changes in the ^{1}H NMR spectrum of **3** (bottom, red) in 1:1 CD_3CN:$CDCl_3$ upon addition of $NaBPh_4$ (second from bottom, yellow). For clarity, the aromatic region has been scaled to a much higher intensity than the aliphatic region.

6.2 DOSY NMR Spectra

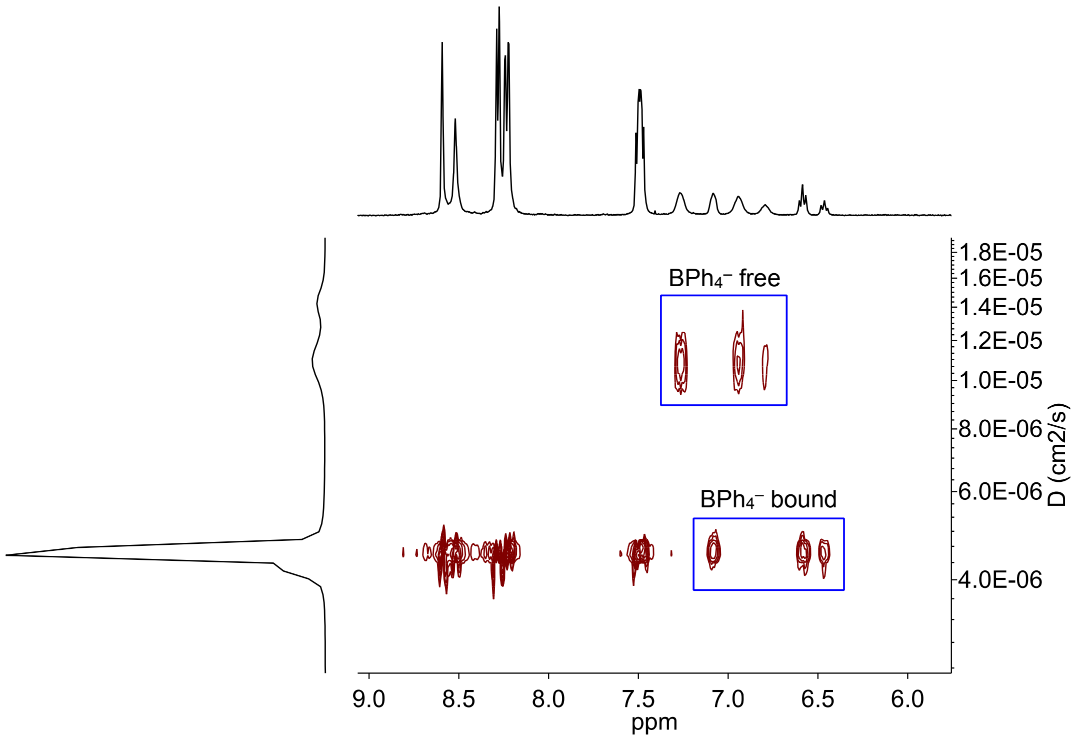

Figure S40. Aromatic region of the DOSY NMR spectrum of **3** in CD_3CN after addition of one equivalent of $NaBPh_4$.

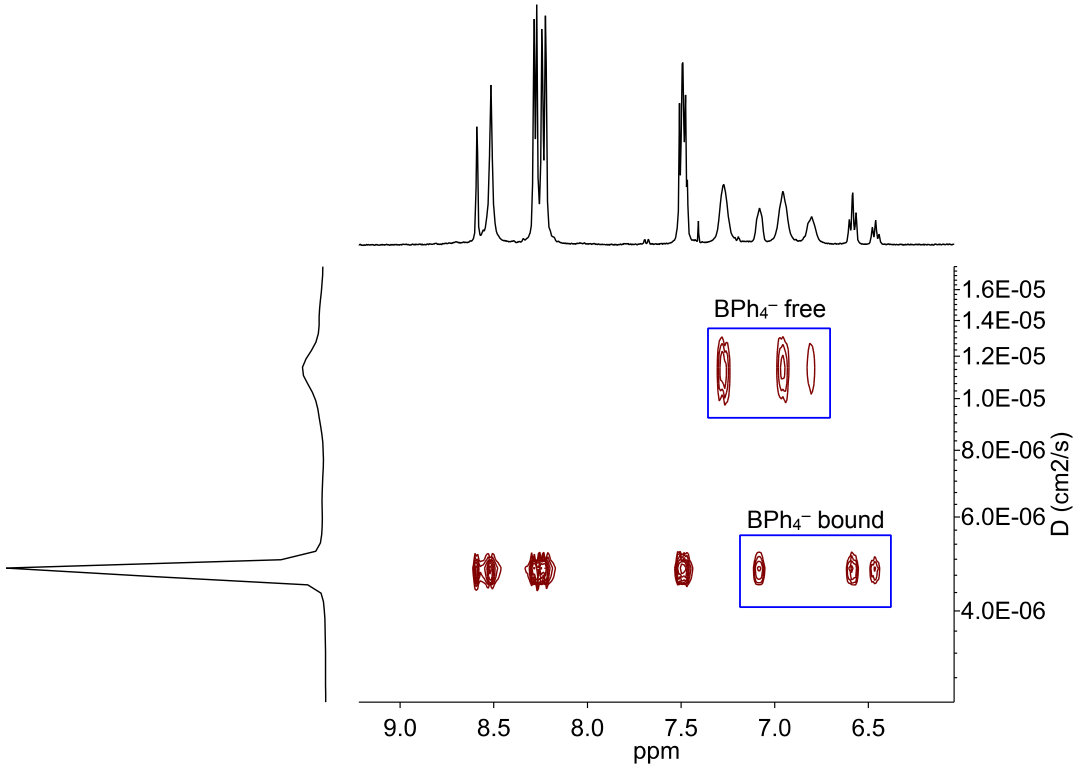

Figure S41. Aromatic region of the DOSY NMR spectrum of **3** in CD_3CN after addition of two equivalents of $NaBPh_4$.

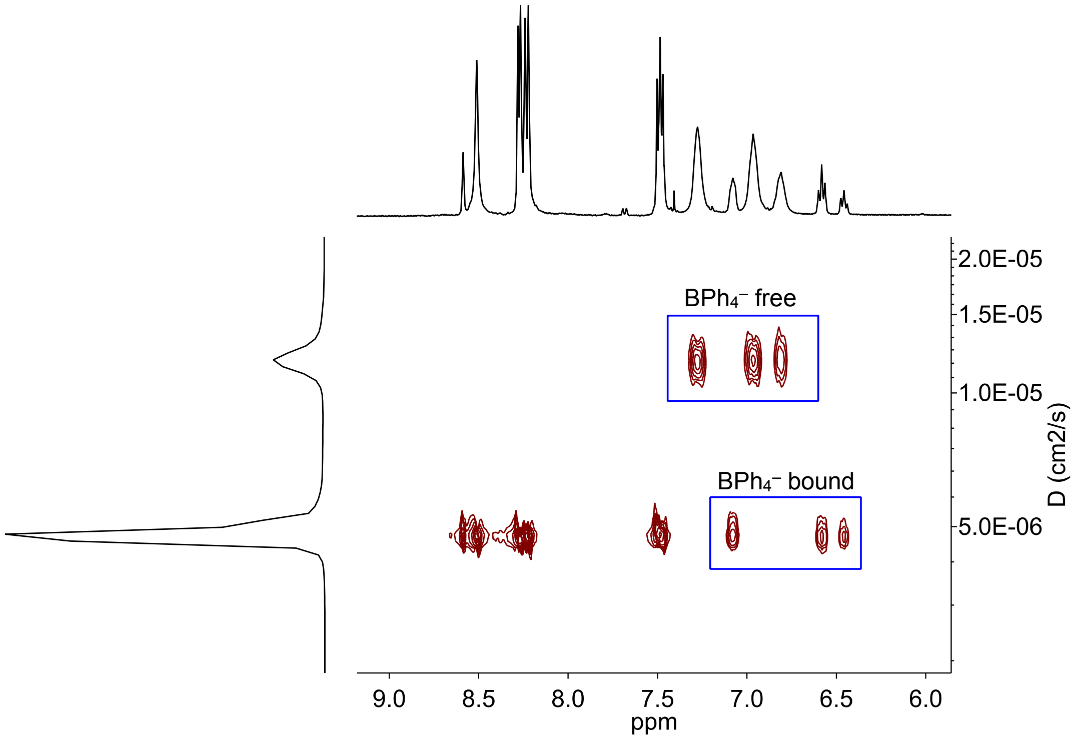

Figure S42. Aromatic region of the DOSY NMR spectrum of **3** in CD_3CN after addition of three equivalents of $NaBPh_4$.

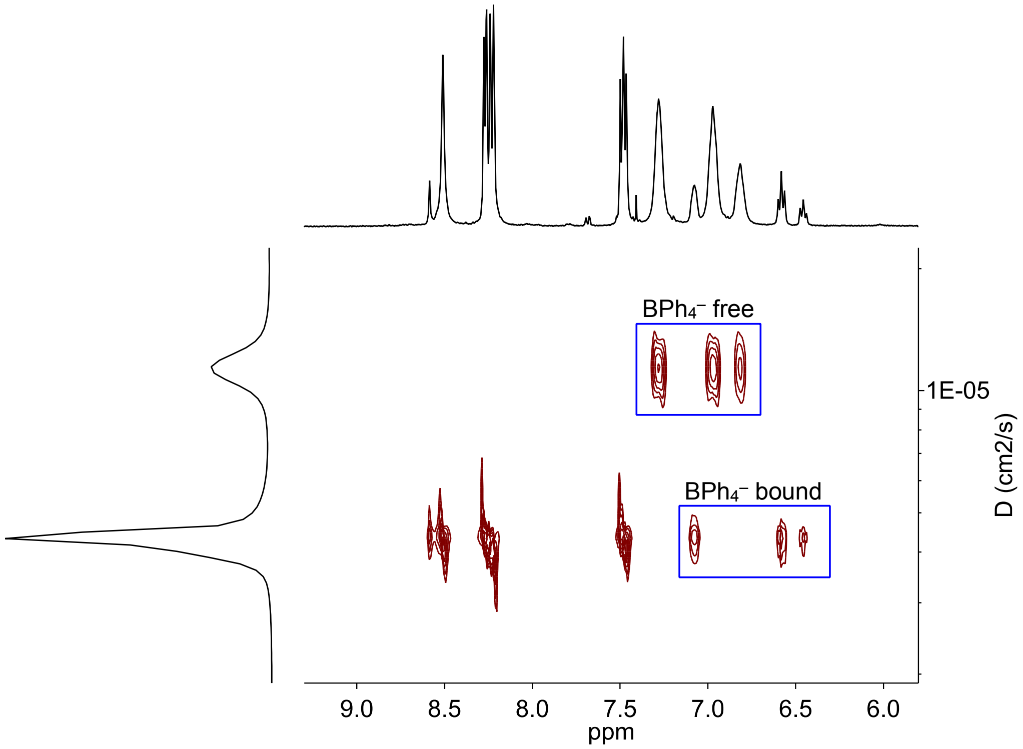

Figure S43. Aromatic region of the DOSY NMR spectrum of **3** in CD_3CN after addition of four equivalents of $NaBPh_4$.

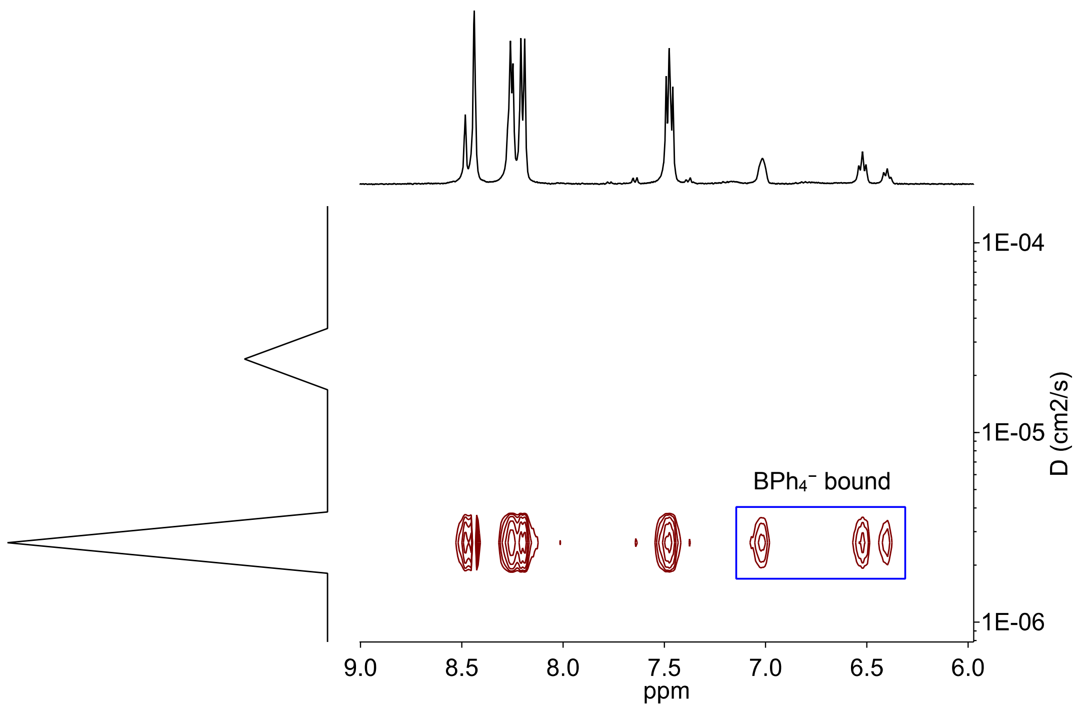

Figure S44. Aromatic region of the DOSY NMR spectrum of **3** in 2:1 CD_3CN:D_2O after addition of one equivalent of $NaBPh_4$. Unbound BPh_4^- anion peaks are barely resolvable due to the low concentration of the species.

6.3 NOESY NMR Spectra

NOE cross-peaks were observed between the protons of the CH_3 groups of the clathrochelate complex and all protons of the bound BPh_4^- anion (Figure S45). Conversely, no NOE cross peaks were observed between the resonances corresponding to unbound BPh_4^- anions and resonances of **3**.

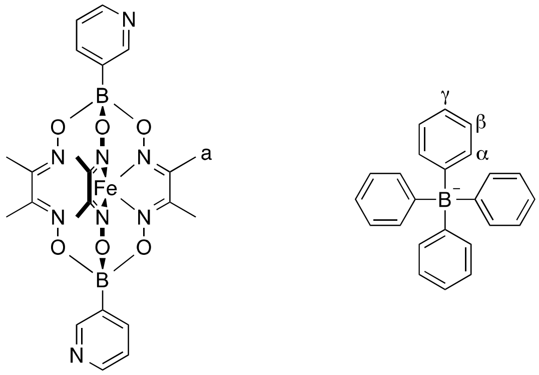

Figure S45. The CH_3 group protons of **3** (a, left) and protons of the BPh_4^- anion (α, β, γ, right) between which NOE cross peaks are observed.

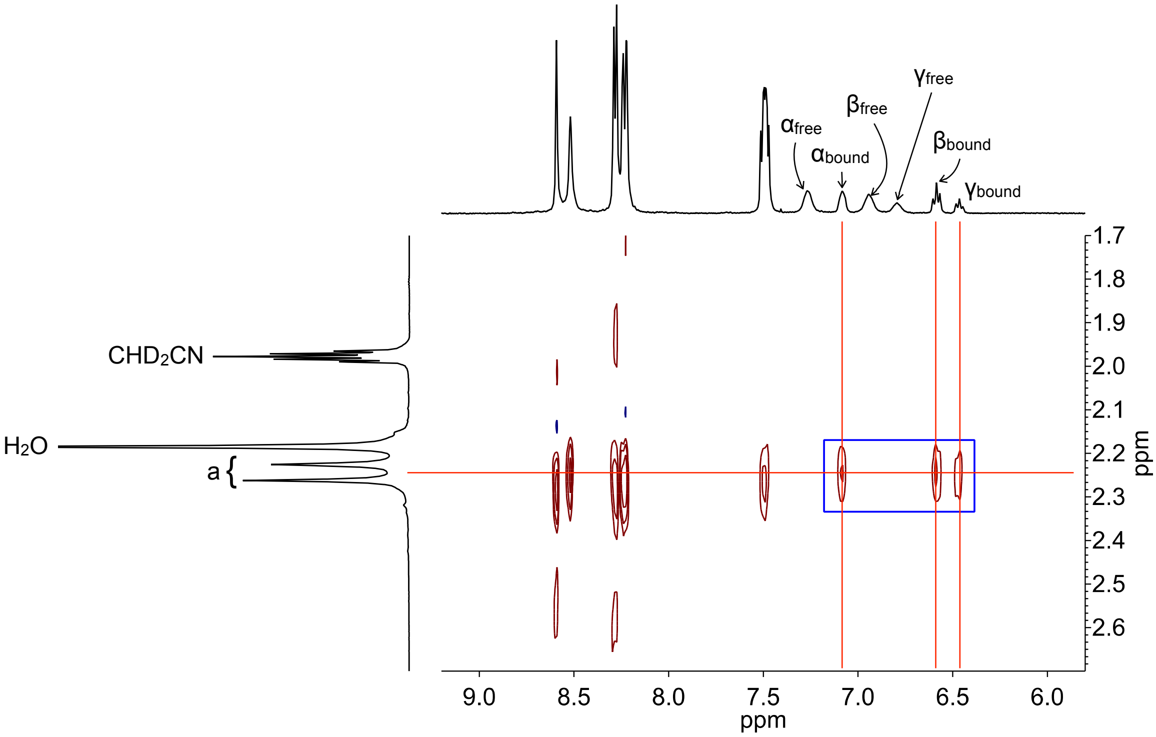

Figure S46. Aromatic/aliphatic region of the NOESY ^{1}H NMR spectrum of **3** in CD_3CN after addition of one equivalent of $NaBPh_4$ showing the NOE cross peaks (blue box).

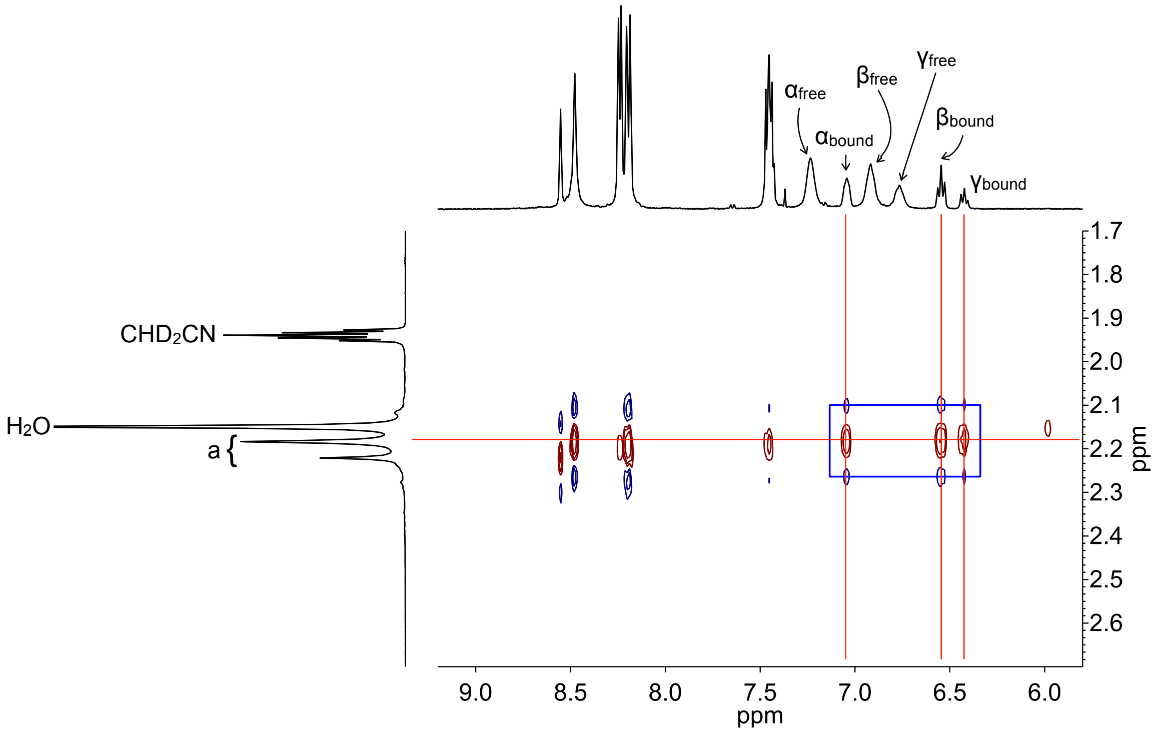

Figure S47. Aromatic/aliphatic region of the NOESY ^{1}H NMR spectrum of **3** in CD_3CN after addition of two equivalents of $NaBPh_4$ showing the NOE cross peaks (blue box).

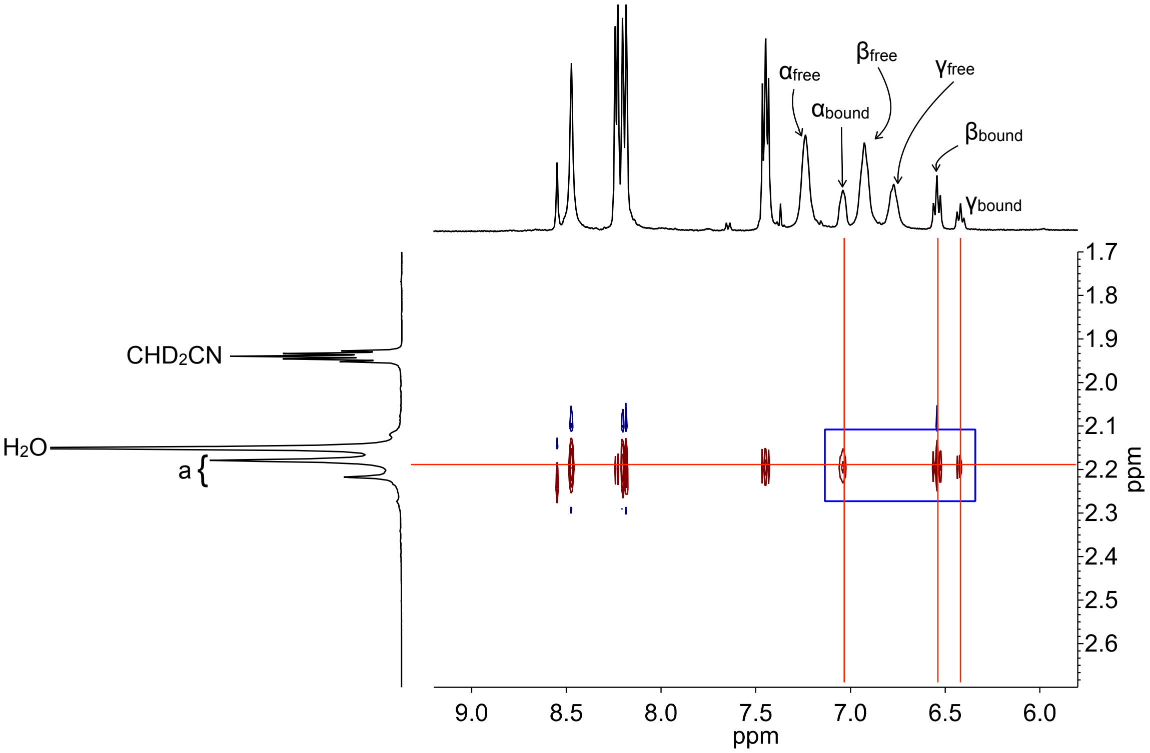

Figure S48. Aromatic/aliphatic region of the NOESY ^{1}H NMR spectrum of **3** in CD_3CN after addition of three equivalents of $NaBPh_4$ showing the NOE cross peaks (blue box).

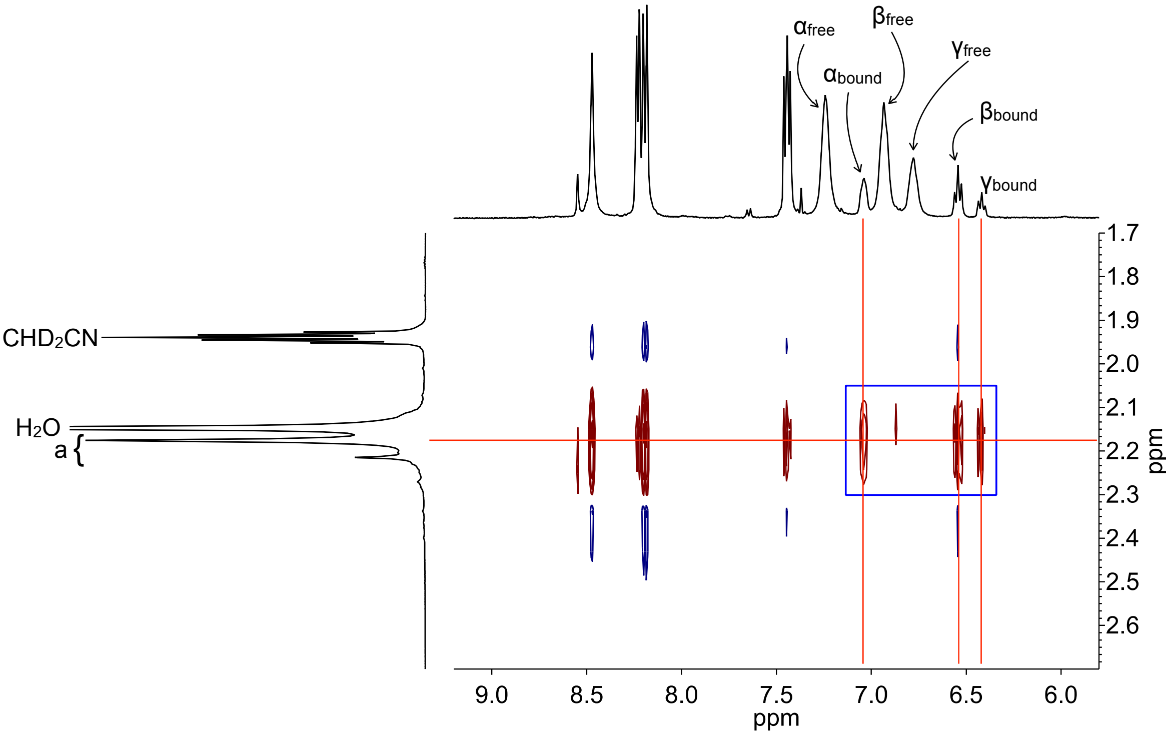

Figure S49. Aromatic/aliphatic region of the NOESY ^{1}H NMR spectrum of **3** in CD_3CN after addition of four equivalents of $NaBPh_4$ showing the NOE cross peaks (blue box).

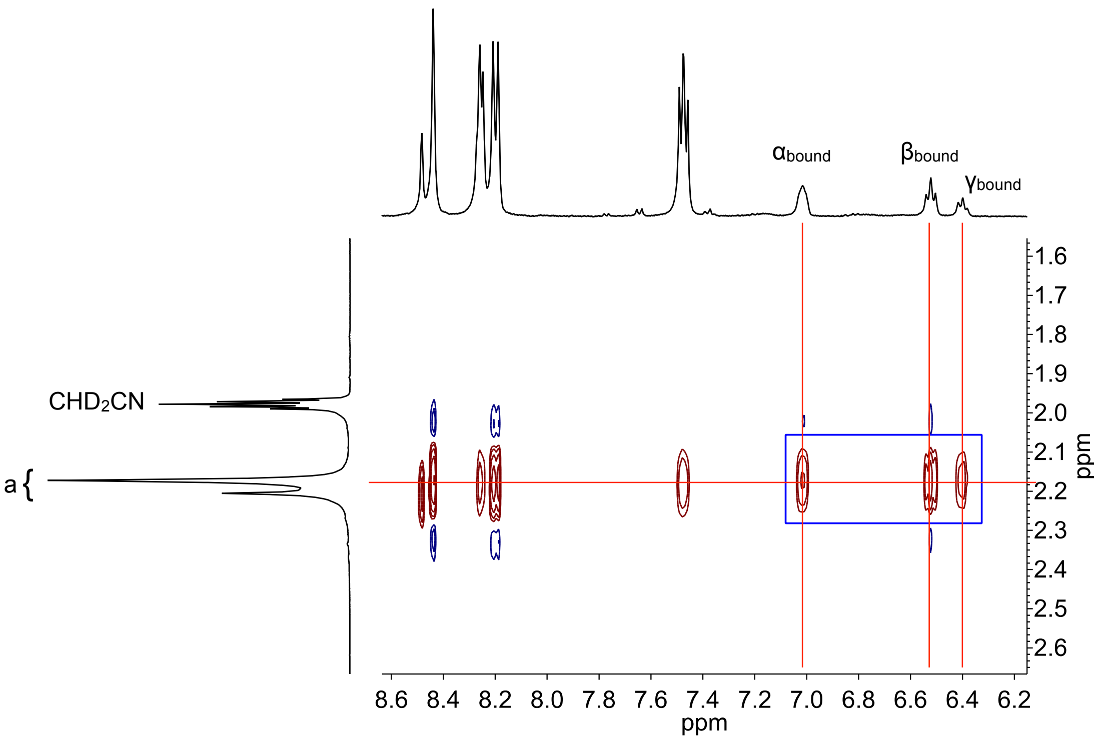

Figure S50. Aromatic/aliphatic region of the NOESY ^{1}H NMR spectrum of **3** in 2:1 CD_3CN:D_2O after addition of one equivalent of $NaBPh_4$ showing the NOE cross peaks (blue box). Unbound BPh_4^- anion peaks are barely resolvable due to the low concentration of the species.

6.4 Calculation of Association Constants

Association constants were calculated by integration of ^{1}H NMR signals of protons α, β and γ corresponding to bound and unbound BPh_4^- anion. The ratio of the integrals of the two species was used to determine their relative concentrations.

6.4.1 Association Constants in CD_3CN

Table S3. Calculation of association constants between **3** and the BPh_4^- anion in CD_3CN.

Equivalents added	*Total sample volume (L)*	*[3] (M)*	*[NaBPh4] (M)*	*Proton*	*Absolute peak intensity*	*[Species] (M)*	K_a (M^{-1})
1	5.10 x 10^{-4}	6.16 x 10^{-4}	6.16 x 10^{-4}	α_{free}	18407.79	3.52 x 10^{-4}	**2.13 x 10^3**
				α_{bound}	13800.58	2.64 x 10^{-4}	
				β_{free}	20873.46	3.56 x 10^{-4}	**2.20 x 10^3**
				β_{bound}	15284.85	2.60 x 10^{-4}	
				γ_{free}	10425.00	3.54 x 10^{-4}	**2.41 x 10^3**
				γ_{bound}	7699.73	2.62 x 10^{-4}	
2	5.20 x 10^{-4}	6.04 x 10^{-4}	1.21 x 10^{-3}	α_{free}	34840.06	8.20 x 10^{-4}	**2.20 x 10^3**
				α_{bound}	16512.00	3.89 x 10^{-4}	
				β_{free}	35256.47	8.14 x 10^{-4}	**2.20 x 10^3**
				β_{bound}	17080.25	3.94 x 10^{-4}	
				γ_{free}	18641.30	8.24 x 10^{-4}	**2.20 x 10^3**
				γ_{bound}	8684.80	3.84 x 10^{-4}	
3	5.30 x 10^{-4}	5.93 x 10^{-4}	1.78 x 10^{-3}	α_{free}	47438.99	1.33 x 10^{-3}	**2.41 x 10^3**
				α_{bound}	16144.56	4.52 x 10^{-4}	
				β_{free}	48382.56	1.33 x 10^{-3}	**2.43 x 10^3**
				β_{bound}	16509.31	4.52 x 10^{-4}	
				γ_{free}	25074.46	1.32 x 10^{-3}	**2.51 x 10^3**
				γ_{bound}	8640.79	4.56 x 10^{-4}	
4	5.40 x 10^{-4}	5.82 x 10^{-4}	2.33 x 10^{-3}	α_{free}	67411.96	1.85 x 10^{-3}	**2.60 x 10^3**
				α_{bound}	17579.68	4.81 x 10^{-4}	
				β_{free}	68635.85	1.84 x 10^{-3}	**2.76 x 10^3**
				β_{bound}	18118.55	4.86 x 10^{-4}	
				γ_{free}	35128.91	1.84 x 10^{-3}	**2.71 x 10^3**
				γ_{bound}	9243.08	4.85 x 10^{-4}	

6.4.2 Association Constants in 2:1 CD_3CN:D_2O

Table S4. Calculation of association constants between **3** and the BPh_4^- anion in 2:1 CD_3CN:D_2O. Small changes in the integration range have significant impacts upon the absolute peak intensity, hence the large error in calculated values. The signals corresponding to protons γ are too weak to be integrated reliably.

Equivalents added	*Total sample volume (L)*	*[3] (M)*	*[NaBPh₄] (M)*	*Proton*	*Absolute peak intensity*	*[Species] (M)*	*K_a (M^{-1})*
0.9	5.09×10^{-4}	2.76×10^{-4}	2.49×10^{-4}	α_{free}	12.06	1.54×10^{-5}	**3.51×10^{5}**
				α_{bound}	182.35	2.33×10^{-4}	
				β_{free}	9	1.15×10^{-5}	**6.79×10^{5}**
				β_{bound}	191.49	2.44×10^{-4}	
1.0	5.10×10^{-4}	2.72×10^{-4}	2.72×10^{-4}	α_{free}	14.78	3.23×10^{-5}	**2.30×10^{5}**
				α_{bound}	109.65	2.39×10^{-4}	
				β_{free}	12.12	2.71×10^{-5}	**3.34×10^{5}**
				β_{bound}	109.53	2.45×10^{-4}	
1.1	5.11×10^{-4}	2.67×10^{-4}	2.94×10^{-4}	α_{free}	16.46	5.15×10^{-5}	**1.90×10^{5}**
				α_{bound}	77.5	2.42×10^{-4}	
				β_{free}	13.05	4.16×10^{-5}	**4.08×10^{5}**
				β_{bound}	79.18	2.52×10^{-4}	
1.2	5.12×10^{-4}	2.63×10^{-4}	3.15×10^{-4}	α_{free}	35.04	7.12×10^{-5}	**1.85×10^{5}**
				α_{bound}	120.27	2.44×10^{-4}	
				β_{free}	30.63	6.47×10^{-5}	**3.19×10^{5}**
				β_{bound}	118.69	2.51×10^{-4}	

7. References

1. A. Technologies, CrysAlis PRO, 2009–2014, Agilent Technologies Ltd, Yarton, Oxfordshire, UK.
2. P. van der Sluis and A. L. Spek, *Acta Crystallogr.* A, 1990, **46**, 194–201.
3. A. J. M. Duisenberg, L. M. J. Kroon-Batenburg and A. M. M. Schreurs, *J. Appl. Crystallogr.*, 2003, **36**, 220–229.
4. automar, release 2.8.0, Marresearch GmbH, Germany, 2011.
5. W. Kabsch, *Acta Crystallogr. D,* 2010, **66**, 125–132.
6. W. Kabsch, *Acta Crystallogr. D,* 2010, **66**, 133–144.
7. C. Vonrhein, C. Flensburg, P. Keller, A. Sharff, O. Smart, W. Paciorek, T. Womack and G. Bricogne, *Acta Crystallogr. D*, 2011, **67**, 293–302.
8. Bruker-Nonius, APEX, SAINT, SADABS and XPREP, Bruker AXS Inc., Madison, Wisconsin, USA, 2013.
9. M. C. Burla, R. Caliandro, M. Camalli, B. Carrozzini, G. L. Cascarano, C. Giacovazzo, M. Mallamo, A. Mazzone, G. Polidori and R. Spagna, *J. Appl. Cryst.*, 2012, **45**, 357−361.
10. G. Sheldrick, *Acta Crystallogr.* A, 2008, **64**, 112–122.
11. L. Palatinus and G. Chapuis, *J. Appl. Crystallogr.*, 2007, **40**, 786–790.
12. C. B. Hübschle, G. M. Sheldrick and B. Dittrich, *J. Appl. Crystallogr.*, 2011, **44**, 1281–1284.
13. M. Pascu, M. Marmier, C. Schouwey, R. Scopelliti, J. J. Holstein, G. Bricogne and K. Severin, *Chem. Eur. J.*, 2014, **20**, 5592–5600.
14. C. Schouwey, J. J. Holstein, R. Scopelliti, K. O. Zhurov, K. O. Nagornov, Y. O. Tsybin, O. S. Smart, G. Bricogne and K. Severin, *Angew. Chem., Int. Ed.*, 2014, DOI: 10.1002/anie.201407144.
15. T. K. Ronson, C. Giri, N. K. Beyeh, A. Minkkinen, F. Topić, J. J. Holstein, K. Rissanen and J. R. Nitschke, *Chem. Eur. J.* 2013, **19**, 3374–3382.
16. G. Bricogne, E. Blanc, M. Brandl, C. Flensburg, P. Keller, P. Paciorek, P. Roversi, A. Sharff, O. Smart, C. Vonrhein and T. Womack, BUSTER version 2.13.0, 2011, Global Phasing Ltd., Cambridge, United Kingdom.
17. http://grade.globalphasing.org.
18. O. S. Smart, T. O. Womack, C. Flensburg, P. Keller, W. Paciorek, A. Sharff, C. Vonrhein and G. Bricogne, *Acta Crystallogr. D*, 2012, **68**, 368–380.

19. A. Thorn, B. Dittrich and G. M. Sheldrick, *Acta Crystallogr.* A, 2012, **68**, 448–451.
20. D. Kratzert, J. J. Holstein and I. Krossing, *J. Appl. Crystallogr.*, *submitted*
21. https://www.xs3.uni-freiburg.de/research/dsr
22. P. van der Sluis and A. L. Spek, *Acta Crystallogr.* A, 1990, **46**, 194–201.
23. A. L. Spek, PLATON: A Multipurpose Crystallographic Tool, 2008, Utrecht University, Utrecht, The Netherlands.
24. G. J. Kleywegt and T. A. Jones, *Acta Crystallog. D*, 1994, **50**, 178–185.
25. W. L. DeLano, The PyMol Molecular Graphics System, DeLano Scientific LLC, San Carlos,CA (USA).
